# MX2 mediates establishment of interferon response profile, regulates XAF1, and can sensitize melanoma cells to targeted therapy

**DOI:** 10.1002/cam4.3846

**Published:** 2021-03-18

**Authors:** Marina Juraleviciute, Jérémie Nsengimana, Julia Newton‐Bishop, Gert J. Hendriks, Ana Slipicevic

**Affiliations:** ^1^ Department of Pathology Oslo University Hospital Oslo Norway; ^2^ Institute for Clinical Medicine Faculty of Medicine University of Oslo Oslo Norway; ^3^ Faculty of Medical Sciences Population Health Sciences Institute Newcastle University Newcastle upon Tyne UK; ^4^ Division of Haematology and Immunology Institute of Medical Research at St James's University of Leeds Leeds UK; ^5^ Department of Cell and Molecular Biology Karolinska Institutet Stockholm Sweden

**Keywords:** melanoma‐specific survival, MX2, STAT1 phosphorylation, XAF1

## Abstract

*MX2* is an interferon inducible gene that is mostly known for its antiviral activity. We have previously demonstrated that *MX2* is also associated with the tumorigenesis process in melanoma. However, it remains unknown which molecular mechanisms are regulated by *MX2* in response to interferon signaling in this disease. Here, we report that MX2 is necessary for the establishment of an interferon‐induced transcriptional profile partially through regulation of STAT1 phosphorylation and other interferon‐related downstream factors, including proapoptotic tumor suppressor XAF1. MX2 and XAF1 expression tightly correlate in both cultured melanoma cell lines and in patient‐derived primary and metastatic tumors, where they also are significantly related with survival. MX2 mediates IFN growth‐inhibitory signals in both XAF1 dependent and independent ways and in a cell type and context‐dependent manner. Higher MX2 expression renders melanoma cells more sensitive to targeted therapy drugs such as vemurafenib and trametinib; however, this effect is XAF1 independent. In summary, we uncovered a new mechanism in the complex regulation of interferon signaling in melanoma that can influence both survival and response to therapy.

## Graphical abstract

MX2 upregulation establishes an interferon‐induced transcriptional profile in melanoma cells. MX2 regulates tumor suppressor XAF1 and sensitizes melanoma cells to targeted therapy drugs such as vemurafenib and trametinib.

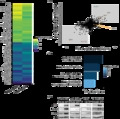

## 1 INTRODUCTION

Cutaneous melanoma arising from epidermal melanocytes is highly lethal skin cancer when it metastasizes. Melanoma genetics and pathogenesis are complex and heterogeneous, and new inherited genetic variation contributing to melanoma susceptibility and development is still being identified.^1^, ^2^, ^3^ Previously reduced risk of melanoma has been correlated to a SNP (rs45430) intronic to interferon response *MX2* (myxovirus resistance 2) gene in genome‐wide association studies.^4^ We have reported that MX2 has tumor‐suppressive activity and is downregulated during the progression of melanoma. Its expression is also a predictor of better patient survival.^5^ It is possible that MX2 downregulation is a result of frequently observed downregulation of interferon signaling in melanoma that ultimately can result in reduced immune cell recruitment and immune recognition of tumor cells.^6^, ^7^ Recently, it has been disclosed that the interferon (IFN) pathway may have a crucial role in melanoma resistance to immunotherapy.^8^, ^9^

IFN effects are mediated by a complex downstream network of hundreds of proteins. Signal transducer and activator of transcription (STAT1) plays an essential role in regulating the transcription of IFN‐stimulated genes (ISGs), including *MX2*.^10^ Upon IFN stimulation, STAT1 is activated by Janus kinases (JAKs) mediated phosphorylation at tyrosine 701, which causes a conformational change of STAT1 and subsequent nuclear translocation. However, it is still unclear what role *MX2 *has in IFN response in melanoma and through which molecular mechanism these effects are exerted.

Another IFN‐regulated gene is *XAF1* (X‐linked inhibitor of apoptosis [XIAP]‐associated factor 1), a tumor suppressor initially identified as XIAP inhibitor.^11^ XAF1 binds to XIAP and interferes with its anti‐caspase activity by sequestering XIAP from cytoplasm to the cell nucleus. However, proapoptotic function of XAF1 is not only XIAP dependent—it has been shown that XAF1 directly binds to p53 and blocks its interaction with MDM2 ubiquitin protein ligase.^12^ Interaction between XAF1 and p53 results in cells favoring apoptosis over cell cycle arrest. XAF1 enhances IFN‐induced apoptosis and profoundly affects IFN‐mediated sensitization of the cells to the proapoptotic actions of TNF‐related apoptosis‐inducing ligand (TRAIL).^13^, ^14^ Furthermore, XAF1‐depleted tumors display an increased resistance to chemotherapeutic drugs.^15^ It has been reported that XAF1 exists in several isoforms and that the full‐length transcript (XAF1A) is often inactivated in several tumors types, including melanoma.^16^ Reduced protein levels of XAF1 have been reported in primary melanoma tumors compared to nevi.^17^

In this study, we have investigated molecular mechanisms regulated by MX2 in melanoma. Our findings, for the first time, show that MX2 is involved in a complex regulatory network of IFN signaling and contributes to STAT1 activation and regulation of XAF1. MX2 mediates the effects of IFN signaling in a cell type and context‐dependent manner that can influence cells sensitivity to MAPK pathway targeted therapies.

## 2 MATERIALS AND METHODS

### 2.1 Cell lines and patient samples

Metastatic melanoma (MM) cell lines were established as described in Flørenes et al.,^18^ and WM melanoma cell lines from the Wistar cell line collection (Philadelphia, PA, USA) were a kind gift of Meenhard Herlyn (cells can be obtained from Rockland Immunochemicals, Inc., PA, USA; catalog numbers are provided in Table S1). Melanoma cells were cultured in RPMI 1640 medium (Bio Whittaker, Verviers, Belgium) supplemented with 5% fetal bovine serum (cat.no F7524, Sigma), 2‐mM l‐glutamine (cat. No. 17‐605E, Lonza). Primary human melanocytes (NHM9, NHM134, and NHM160) were obtained as previously described ^19^ and cultured in 254CF melanocyte media (cat. No. M254CF500) purchased from Gibco Life Technologies (California, USA), supplemented with calcium chloride, HMGS‐2 (human melanocytes growth supplement‐2) (cat. No. S0165, Gibco), and 10‐ng/ml PMA. All cells were maintained at 37°C in a humidified 5% CO_2_ atmosphere.

Melanoma lymph node metastases were obtained from patients operated at the Norwegian Radium Hospital, Oslo University Hospital. The study design was approved by Norway Regional Committee for Medical and Health Research Ethics (approval number 2015/2434). Written informed consent was obtained from all patients included in the study.

### 2.2 Reagents

Vemurafenib (cat. No. S1267) and trametinib (cat. No. S2673) were purchased from Selleck Chemicals (Houston, TX, USA) and reconstituted in DMSO according to manufacturer's recommendations. Vemurafenib was used at 2‐µM concentration and trametinib at 100‐nM concentration in all experiments involving these reagents. Recombinant human IFNα (cat. No. SRP4596) was purchased from Sigma‐Aldrich. Cells were incubated with 1000‐ or 25 000‐IU/ml final concentration of IFNα. Doxycycline (cat. No. D9891‐1G, Sigma) was used at 500‐ng/ml concentration for 24 h to induce MX2/GFP expression in transduced normal human melanocytes. Control groups were exposed to the same amounts of drug vehicle DMSO or H_2_O as treatment groups.

### 2.3 Cell viability assay

For viability assays cells were seeded into 96‐well plates at density 5000 cells per well. After treatments cell viability was determined using CellTiter‐Glo luminescent viability assay (cat. No. G7570) purchased from Promega and following manufacturer's protocol. Luminescence was recorded with Fluoroskan Ascent FL luminometer (Thermo Fisher Scientific).

### 2.4 siRNA knockdown

For RNA and protein analyses cells were plated into six‐well plates at density of 2 × 10^5^ per well 24 h before transfection. For Incucyte proliferation and CellTiter‐Glo viability assays cells were seeded into 96‐well plates at density 5000 cells per well. Cells were transfected with siRNAs targeting *MX2* and *XAF1*. *MX2* #1 siRNA targeting sequence 5′‐GGAAACAGGAGCCAACCAAtt‐3′ (cat. No. AM16706, ID 11695; Ambion, Life Technologies) and #2—targeting sequence 5′‐GGAUUUUAAAAACUGGGUAtt‐3′ (cat. No. AM16708, ID 11785; Ambion, Life Technologies). *XAF1* siRNA targeting sequence 5′‐CCAUAUGGGUAAAUGUUGUtt‐3′ (cat. No. AM16704, ID 140389; Ambion, Life Technologies). Nontargeting siRNA (cat. No. 4390843; Ambion, Life Technologies) was used as a negative control. Transfections performed using Lipofectamine RNAiMAX transfection reagent (cat. No. 13778150, Invitrogen) in Opti‐MEM reduced serum media (ref. 51985–026, Gibco) and following manufacturer's protocol. Transfections lasted for 24 before addition of 25000‐ IU/ml of IFNα and vemurafenib/trametinib treatments and for 72 h before addition of 1000‐IU/ml IFNα. *MX2* and *XAF1* siRNAs were used at 40‐nM final concentration before IFNα treatments and at 20‐nM concentration before vemurafenib and trametinib treatments.

### 2.5 Incucyte growth rate assessment

Cells were seeded into 96‐well plates at density 5000 cells per well. Cell proliferation after XAF1 knockdown was measured for 48 or 72 h by a confluence assay using IncuCyte^TM^ FLR (Essen Instruments, Ann Arbor, MI) live cell imaging system. Cell confluence after MX2/XAF1 knockdown followed by 25000‐IU/mL IFNα treatment was measured immediately after IFNα addition and 48 h later. Cell growth rate was determined by normalizing cell confluence at a given time to the corresponding initial time point. Relative growth represents the ratios between growth rates of IFNα‐treated and corresponding untreated samples. *N* = 3 independent experiments were performed with two to three technical replicates per each group. Data are presented as a mean value of three independent experiments ± SD.

### 2.6 Cytoplasmic and nuclear fractionation

NE‐PER Nuclear and Cytoplasmic Extraction Reagent kit (cat. No. 78833; Thermo Fisher Scientific) was used to isolate cytoplasmic and nuclear proteins. Isolation was performed according to manufacturer's instructions. Halt Protease Inhibitor Cocktail (cat. No. 87785; Thermo Fisher Scientific) was added to the CER I and NER extraction reagents before use.

### 2.7 Quantitative real‐time PCR

Total RNA was extracted from cell samples and tissues using a NucleoSpin RNA extraction kit (ref. 740955.250; Macherey‐Nagel, Duren, Germany) according to manufacturer's instruction. Reverse transcription reactions were performed with SuperScript IV VILO cDNA Synthesis KIT (cat. No. 11756050; Invitrogen) using random primers and following the manufacturer's protocol. Total amount of 0.5‐µg RNA used in 20‐µl of reaction mixture. Real‐time PCR analyses were performed using TaqMan Fast Advanced Master Mix (2×) (cat. No. 4444554; Applied Biosystems) and TaqMan Gene Expression Assays (*MX2*—Hs01550811_m1; *GUSB*—HS99999908_m1; *XAF1*—Hs01550142_m1; *DSG2*—Hs00937265_m1, Applied Biosystems). Twenty microliters of PCR mixture contained 0.2‐µl cDNA, 250‐nM TaqMan probe, and 900 nM of each primer. RT‐qPCR reactions were performed on a QuantStudio^TM^ 5 Real‐Time PCR system (Applied Biosystems, Thermo Fisher Scientific) running the following program^1^: enzyme activation at 95°C for 20 s and ^2^ 40 cycles of PCR at 95°C for 1 s and 60°C for 20 s. Relative transcript expression levels were normalized against a housekeeping gene beta‐glucuronidase *GUSB* and calculated using a comparative Ct method.

### 2.8 Immunoblotting

Cells were scraped from monolayer, washed once in 1× PBS, and lysed in ice‐cold NP‐40 lysis buffer (1% NP‐40, 10% glycerol, 20‐mM Tris‐HCl [pH 7.5], 137‐mM NaCl, 100‐mM NaF) supplemented with phosphatase inhibitor (cat. No. 4906837001, Roche Diagnostics) and protease inhibitor (cat. No. 4693124001, Roche Diagnostics). Bio‐Rad Protein Assay Dye Reagent Concentrate (cat. No. 500–0006, Bio‐Rad) was used according to manufacturer's instructions to quantify proteins in the lysates and 15 µg of proteins per lane was resolved on 4%−20% (cat. No. 5678094, Bio‐Rad) or 10% (cat. No. 5678034, Bio‐Rad) gels by SDS‐PAGE electrophoresis. Proteins were then transferred to the Immobilon‐P PVDF membrane (cat. No. IPVH00010; Merck Millipore) using Trans Blot Turbo transfer system (Bio‐Rad). Membranes were cut, blocked in 5% nonfat milk in TBST (150‐mM NaCl, 20‐mM Tris‐HCl, [pH 7.5], 0.01% Tween‐20) and incubated with primary antibodies overnight at 4°C. Then, membranes were washed 15 min in TBST, hybridized with HRP‐conjugated appropriate secondary antibodies for 1 h at room temperature, and washed again 15 min in TBST. Visualization of proteins was performed using SuperSignal West Dura Chemiluminescence kit (cat. No. 34075, Thermo Scientific™) on a G:BOX (Syngene).

Cell Signaling (Danvers, MA, USA): XAF1 (#13805) 1:1000, H3 (#4499) 1:3000, GAPDH (#2188) 1:2000, β‐tubulin (#15115) 1:2000, β‐actin (#4967) 1:1000, IRF1 (#8478) 1:500, phospho‐STAT1 Y701 (#9167) 1:2000, STAT1 (#14994) 1:2000, Cleaved Caspase 3 (#9664) 1:1000, phospho‐ERK 1/2 (#4370) 1:20000, ERK 1/2 (#4695) 1:20,000, phospho‐AKT s473 (#4060) 1:2000, AKT (#9272) 1:2000; Novus Biologicals (Littleton, CO, USA): MX2 (NBP1‐81018) 1:2000. Secondary antibodies were purchased from Promega (Madison, WI, USA): anti‐rabbit (W4011) 1:2000, anti‐mouse (W4021) 1:2000. Immunoblotting was performed at least twice with independent sets of lysates. All original immunoblots used for the study are provided in Figures [Link], [Link], [Link], [Link], [Link], [Link], [Link].

### 2.9 Generation of stable lines overexpressing MX2 and GFP

Cell lines were established using the same vectors and reagents and following procedures as described in a publication.^5^ Briefly, expression constructs were generated by recombining entry clones pENTR1A containing *MX2* or *GFP* cDNA into the destination vectors. Destination vector pLenti‐CMV‐Puro‐DEST (w118‐1) was used to achieve constitutive MX2/GFP expression in melanoma cell lines; pCW57.1 vector was utilized to obtain doxycycline inducible MX2/GFP overexpression in normal human melanocytes. HEK293T cells were transfected with MX2 or GFP expression vectors and lentiviral packaging plasmids (pCMV‐VSV‐G and pCMV‐ΔR8.2). Lentiviral particles were harvested every 24 h for 3 days, cleared from cellular debris, precipitated, and concentrated by resuspending in 1/10 of original volume in cold PBS. For transduction, cells were overlayed overnight with lentivirus containing 8‐µg/ml polybrene. Then, melanocytes were selected using 1 µg/ml and melanoma cells—2‐μg/ml puromycin.

### 2.10 RNA sequencing and analysis

Libraries were prepared using 10 µg extracted RNA and the TruSeq library preparation kit (Illumina) and sequenced on a NextSeq500 instrument (Illumina) after which 435 M reads were obtained. The resulting reads were aligned to hg38 using STAR version 2.7 ^20^ and count tables were produced using the feature Counts function from the Rsubread package (version 2.0.1).^21^ Differential expression analysis was performed using DEseq2 (version 1.26.0).^22^ Log fold‐change shrinkage was performed using DEseq2 and the adaptive shrinkage estimator from the R package “ashr” (version 2.2–39).^23^ RNA sequencing data are available through the gene expression omnibus (ID GSE168102). GO analysis was conducted with GOrilla.^24^, ^25^ Data were visualized using R and ggplot2.^26^, ^27^

### 2.11 Transcriptomic data

Generation and preprocessing of gene expression data from 703 formalin fixed tumors of the Leeds Melanoma Cohort (LMC, accession number EGAS00001002922) was described elsewhere.^28^ The LMC is a population based cohort of primary cutaneous melanoma. Gene expression data from MMs in The Cancer Genome Atlas (TCGA) (Firehose Legacy) was downloaded from c‐bioportal (https://www.cbioportal.org/). Expression data for *MX2* and *XAF1* in melanoma cell cultures were downloaded from the Cancer Cell Line Encyclopedia (https://portals.broadinstitute.org/ccle).

### 2.12 Statistical analyses

Statistical analysis was performed applying SPSS package Version 18 (SPSS inc., Chicago, IL) and STATA Version 14 (StataCorp, College Station, TX). Association between *MX2* and *XAF1* genes in various datasets was tested by applying Pearson correlation and scatterplots. Kaplan–Meier curves, log rank tests, and Cox proportional‐hazards regression were used to analyze survival after dichotomizing the expression in upregulation and downregulation by median split. Overall survival (OS) was derived in the TCGA dataset while melanoma‐specific survival (MSS) was derived in the LMC dataset. Significance of in vitro results was assessed using Welch's *t* test on log transformed data when comparing two groups or one‐way ANOVA with Tukey's multiple comparison test after log transformation of the data when comparing more than two groups. Proliferation, cell growth, and viability experiments were performed three times independently (*n* = 3) with three technical replicates per treatment group, unless stated otherwise.

## 3 RESULTS

### 3.1 MX2 upregulation leads to induction of IFN signaling signature

**FIGURE 1:**
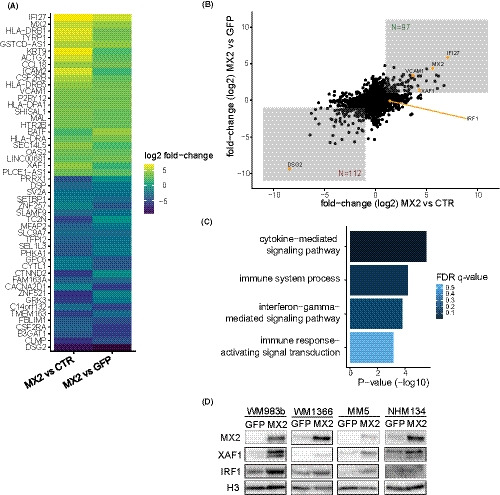
MX2 upregulation induces ISGs expression. (A) Heatmap of top 50 differentially expressed genes between MX2‐overexpressing WM983b melanoma cells and untreated control cells (left row) and between MX2‐overexpressing and GFP‐overexpressing cells (right row). Top 50 genes, upregulated (green) and downregulated (blue), were selected based on their average fold changes while requiring at least two‐fold upregulation or downregulation as well as adjusted *p* values below 0.05 for both comparisons. (B) Scatter plot of differential gene expression between MX2 overexpressing and control cells (CTR/GFP). Boxes indicate genes that are at least two‐fold upregulated or downregulated and with adjusted *p* values below 0.05 for both comparisons. (C) GO enrichment analysis of biological processes for differentially expressed genes between MX2 overexpression against both control and GFP. (D) Validation of RNA‐seq transcriptome analysis by immunoblotting in GFP or MX2 overexpressing WM983b and in additional melanoma cell lines and normal human melanocytes (NHM). MX2/GFP protein expression in NHM was induced by 500‐ng/ml doxycycline treatment for 24 h. H3 was used as a loading control

To identify downstream effectors and molecular mechanisms mediating MX2 tumor growth suppression, we performed RNA‐seq of MX2 overexpressing WM983b melanoma cells. The analysis identified 213 differentially expressed genes between MX2 WM983b‐overexpressing and both GFP‐overexpressing and untransduced control conditions (Table S2), and a heatmap of 50 most differentially expressed genes is displayed in Figure 1A. Among the most significantly upregulated genes were multiple IFN‐regulated genes, including IFN alpha inducible protein 27 (*IFI27*), vascular cell adhesion molecule 1 (*VCAM*‐*1*), and XIAP‐associated factor 1 (*XAF1*) (Figure 1B). The most downregulated gene was desmoglein 2 (*DSG2*). GO analysis confirmed that IFN and immune pathways are significantly enriched in MX2 overexpressing cells (Figure 1C). Interestingly, IFN regulatory factor 1 (*IRF1*), a transcription factor rapidly induced in response to IFN, was significantly upregulated only in MX2‐overexpressing WM983b cells compared to untransduced control cells, suggesting that general antiviral response to lentiviral transduction should be considered when interpreting the results.

To validate these RNA‐seq data, we assessed protein levels of XAF1 and IRF1 in the MX2‐ and GFP‐overexpressing cells since both conditions have undergone viral transduction. In support of RNA‐seq data, we observed that XAF1 protein was upregulated in MX2 overexpressing WM983b cells. Surprisingly, we also detected upregulation of IRF1, suggesting that there is a partial discrepancy between RNA and protein regulation and that MX2 regulatory influence cannot be excluded. Investigation of additional constitutively MX2‐/GFP‐overexpressing melanoma cell lines and normal human melanocytes with doxycycline induced MX2/GFP expression showed similar results (Figure 1D). We also confirmed downregulation of *DSG2* using qRT‐PCR (Figure [Link], [Link]).

### 3.2 MX2 levels affect STAT1 phosphorylation

**FIGURE 2:**
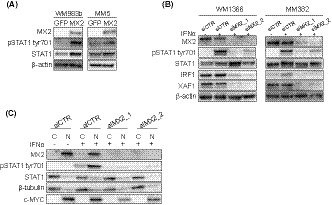
MX2 mediates STAT1 activation. (A) Immunoblot analysis of STAT1 protein phosphorylation at tyr701 and total expression in GFP‐ and MX2‐overexpressing WM983b and MM5 melanoma cells. β‐actin used as a loading control. (B) MX2 was downregulated with two *MX2* targeting siRNAs for 72 h followed by 90‐min treatment with 1000‐IU/ml IFNα in WM1366 and MM382 melanoma cells. Expression of ISGs and STAT1 was assessed by immunoblotting. Control cells (siCTR) were treated with a negative scrambled control siRNA. β‐actin used as a loading control. (C) WM1366 cells were treated as in (B) followed by cytoplasmic and nuclear protein separation. Localization of both MX2 and STAT1 was examined by immunoblotting. β‐tubulin used as a loading control for cytoplasmic proteins, c‐MYC—nuclear

IFN signaling signature is mainly established through the recruitment of STAT transcription factors that activate genes containing IFN‐stimulated response elements (ISREs), such as *XAF1*.^29^ Since we observed higher expression of ISGs in MX2 overexpressing cell lines, we asked whether this could be the result of increased STAT1 signaling pathway activation. Indeed, as shown in Figure 2A, we observed an increase in the protein level of STAT1 and increased phosphorylation of STAT1 regulatory residue tyr701 in WM983b‐MX2 and MM5‐MX2 cells compared to control GFP cells.

To further investigate MX2 and STAT1 relationship, we knocked down MX2 using siRNA in WM1366*^NRASmut^* and M382*^BRAFmut^* cells endogenously expressing MX2 protein. After 72 h, cells were treated with IFNα for 90 min, harvested, and expression of ISGs was assessed by western blotting. In control cells of both lines, exposure to IFNα led to a marked increase of phosphorylated STAT1 at tyr701 and a slight increase of IRF1, which are early response genes, while MX2 and XAF1 levels remained unchanged due to the relatively short incubation time with IFNα (Figure 2B). Interestingly, MX2 knockdown led to strong impairment of STAT1 phosphorylation and activation, suggesting that MX2 is a necessary factor for IFNα‐induced STAT1 activation. Furthermore, MX2 knockdown also led to a clear decrease of IRF1 and XAF1 protein expression compared to unstimulated controls suggesting that it can be involved in their direct regulation irrespectively of IFNα.

It has previously been reported that MX2 can be located at the cytoplasmic side of the nuclear envelope where it is involved in nuclear import.^30^ Since following its activation STAT1 is translocated to the nuclei, we also asked if MX2 might facilitate its translocation. We first knocked down MX2 in endogenously MX2 protein expressing WM1366*^NRASmut^* cells using siRNA for 72 h and then treated them with 1000‐IU/ml IFNα for 90 min. Cytosolic and nuclear cell fractions were extracted and assessed by western blotting. As seen in Figure 2C, IFNα induced STAT1 phosphorylation at tyr701 in control cells, which was detected both in cytoplasmic and nuclear fractions, but as expected, an increase was seen in the nuclear fraction. STAT1 phosphorylation was again severely impaired in cells where MX2 was reduced. However, we detected a weak phosphorylated STAT1 signal in the nucleus and the general distribution remained the same, suggesting that STAT1 translocation could still occur when MX2 is downregulated.

Of note, previously we have demonstrated that MX2 knockdown downregulates expression of multiple nuclear genes,^5^ including Lamin B1 and α‐tubulin, which are commonly used as loading controls for nuclear proteins in fractionation experiments. Therefore, in our study, we chose c‐MYC as a loading control for nuclear fraction of proteins, since it shows nuclear distribution and is not affected by MX2 downregulation in melanoma cells.

### 3.3 MX2 regulates XAF1 expression

**FIGURE 3:**
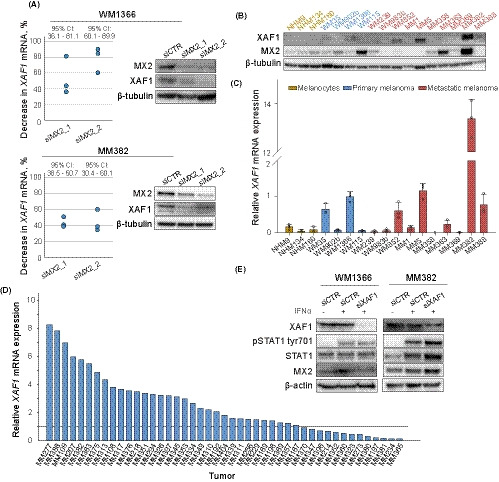
Characterization of XAF1 expression. (A) WM1366 and MM382 cells were transfected with control siRNA (siCTR) or *MX2* specific siRNAs (siMX2_1 and siMX2_2). Forty‐eight hours post transfection, proteins and RNA were extracted and analyzed by immunoblotting and RT‐qPCR, respectively. Dot plot depicts % decrease of *XAF1* mRNA expression compared to siCTR. Each dot represents mean value of an independent experiment (*n* = 3). β‐tubulin was used for protein loading control. (B) Immunoblot analysis of XAF1 and MX2 protein expression in normal human melanocytes (NHM), primary and metastatic melanoma lines (β‐tubulin used as a loading control; MX2 immunoblot has been previously published in Juraleviciute et al.^5^) and (C) *XAF1* mRNA expression analysis in the same panel of cell lines. Bar represents mean value + SD (*n* = 3), and circles depict mean value of each independent experiment. *XAF1* mRNA expression is normalized to primary melanoma WM1366 cell line. (D) *XAF1* mRNA expression in melanoma tumor samples derived from lymph node metastases. Tumors expressing lower *XAF1* mRNA levels compared to primary WM1366 are below the line. (E) Expression of ISGs and STAT1 in WM1366 and MM382 melanoma cells after 72 h of XAF1 downregulation with siRNA followed by 90‐min treatment with 1000‐IU/mL IFNα (β‐actin used as a loading control)

Since XAF1 was constitutively expressed in MX2 expressing WM1366*^NRASmut^* and M382*^BRAFmut^* cells, we used them to further investigate XAF1 dependency on MX2. As seen in Figure 3A, siRNA‐mediated MX2 knockdown led to a significant decrease of both RNA and protein levels of XAF1 in both cell lines, suggesting an IFN‐independent regulatory link between XAF1 and MX2. We also examined *XAF1* mRNA and protein expression in melanocytes and melanoma cell line panel previously investigated for *MX2* expression. Immunoblotting revealed differential XAF1 protein expression that correlated well with the mRNA levels (Figure 3B,C). Interestingly, low *XAF1* expression was seen in normal human melanocytes, while it was present in two primary and four metastatic lines. Variable expression of *XAF1* RNA was also observed in our panel of 45 patient‐derived lymph node MM samples (Figure 3D) that significantly correlated with previously reported *MX2* RNA expression^5^ (Figure [Link], [Link]). The correlation between *XAF1* and *MX2* mRNA was also high in cell lines (*R* = 0.47, *p* = 0.06, and data not shown), but sample size was too small to achieve statistical significance. The analysis of 55 established melanoma cell lines of primary and metastatic origin from the Cancer Cell Line Encyclopedia ^31^, ^32^ showed a comparable but more significant correlation (*R* = 0.55, *p* < 0.001) (Figure [Link], [Link]) further supporting their regulatory relationship.

Since our result show that MX2 can regulate XAF1 expression, we also tested whether XAF1 can form a feedback loop with MX2 as it was proposed for other proteins.^33^ siRNA‐mediated knockdown of XAF1 followed by the IFNα treatment did not alter MX2 expression or STAT1 phosphorylation status placing XAF1 as a downstream effector of these proteins (Figure 3E). Furthermore, when we checked tyr701 phosphorylation of STAT1 in the same cell line panel as above, we did not observe any clear association with XAF1 protein expression (Figure [Link], [Link]) again suggesting that XAF1 expression might be regulated by MX2 independently of STAT1.

### 3.4 MX2 and XAF1 mediate IFN growth‐inhibitory effects in a cell type‐specific manner

Previously, it has been reported that MX2 overexpression leads to growth inhibition of melanoma cells in a cell type‐specific manner.^5^ Since we here have identified XAF1 as an effector downstream of MX2, and XAF1 has previously been shown to inhibit tumor growth and mediate apoptotic effects in several cancers,^34^, ^35^, ^36^ we asked if XAF1 can have a similar function in melanoma cells.

**FIGURE 4:**
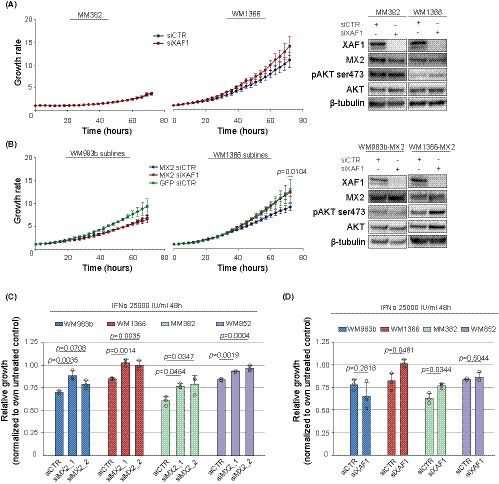
MX2 can mediate growth inhibition through XAF1 regulation. (A) Proliferation assessment (left panel) by Incucyte and immunoblot analysis of protein expression (right panel) in cells treated with scrambled RNA (siCTR) and *XAF1* specific siRNA for 72 h. Growth rates were calculated by normalizing cell occupied surface area at a given time point to initial. Each point of growth curves represents mean value ± SD (*n* = 3). Comparison of siCTR and siXAF1 growth rates at 72‐h time point was performed by Welch's *t* test after log transformation of the data. (B) GFP‐overexpressing cells were transfected with scrambled RNA (siCTR), while MX2‐overexpressing WM983b and WM1366 cells were treated with siCTR and siXAF1, and proliferation, protein expression, and statistical significance were assessed as in (A). (C) Melanoma cells were transfected with siMX2 and (D) siXAF1 for 24 h followed by confluence assessment on Incucyte system right after 25,000‐IU/mL IFNα addition and 48 h later. Relative growth represents the ratios between growth rates of IFNα treated and respective IFNα untreated controls at 48‐h time point. Each circle on histogram shows mean value of an independent experiment and each bar represents the mean + SD (*n* = 3). Statistical significance was assessed using one‐way ANOVA with Tukey's multiple comparison test in (C) and Welch's *t* test in (D) after log transformation of data

We first tested the effects of XAF1 siRNA knockdown (Figure 4A, left panel) on the proliferation of XAF1 expressing melanoma cells over a 72 h period using Incucyte system. Interestingly, we did not observe any proliferation effects of the knockdown in M382*^BRAFmut^* cells. In the WM1366*^NRASmut^* cells, XAF1 depletion increased cell growth; however, the effect was not strong enough to reach statistical significance. Still, the representation of experimentally paired data in the before–after graph (Figure [Link], [Link]) clearly shows an increase in cell proliferation upon XAF1 downregulation. Furthermore, we performed XAF1 knockdown in additional *BRAF* and *NRAS* mutant melanoma cultures (Figure [Link], [Link]) and observed the same trend. XAF1 depletion promoted cell growth in both *NRAS* mutant cultures—WM852—and in‐house established MM388, suggesting that XAF1 effects are cell type and context dependent.

Since previously it has been shown that MX2 overexpression influences activation of the AKT pathway, we also examined if XAF1 could be involved in this regulation. Interestingly, in WM1366 cells, downregulation of XAF1 led to a weak increase of phosphorylated AKT at ser473, while in MM382, it remained unchanged (Figure 4A, right panel); MAPK pathway was not affected in any of the cell lines (Figure [Link], [Link]).

Next, we tested if MX2 growth‐inhibitory effects are mediated by XAF1 in MX2 overexpressing WM983b and WM1366 cells. Downregulation of XAF1 did not show any effects in *BRAF* mutant WM983b‐MX2 cells (Figure 4B, left panel). However, in *NRAS* mutant WM1366‐MX2 cells, XAF1 downregulation restored proliferation back to levels observed in GFP‐overexpressing control cells. Also, in WM1366‐MX2 cells, XAF1 downregulation led to a slight increase in levels of both total and phosphorylated AKT (Figure 4B, right panel).

IFNα is used as an adjuvant treatment for melanoma and is known to exert proliferation modulatory effects on cells of different tumor types. To elucidate the contribution of MX2 and XAF1 to the growth‐inhibitory effect of IFNα, we investigated the proliferation of two *BRAF* and two *NRAS* mutant melanoma cultures after either MX2 or XAF1 downregulation followed by 48‐h IFNα incubation (protein expression assessed in Figure S3D,E). While IFNα treatment reduced the growth of all cell lines between 20% and 40%, this effect was significantly decreased when MX2 induction by IFNα was prevented (Figure 4C). Interestingly, downregulation of XAF1 during IFNα treatment and in the presence of MX2 could only reverse IFNα inhibitory effects in WM1366 and MM382 cells (Figure 4D). This suggests that in the WM983b and WM852 cells, MX2 is mediating IFNα growth‐inhibitory effect independently of XAF1 while in WM1366 and MM382 cells XAF1 is necessary, highlighting that their function is cell context dependent.

### 3.5 XAF1 is associated with survival in primary and metastatic melanoma cohorts

**FIGURE 5:**
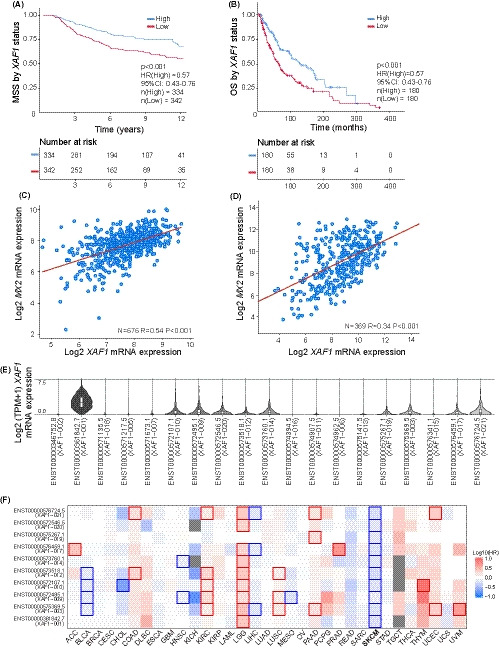
XAF1 expression is associated with a better melanoma survival. (A) Kaplan–Meier melanoma‐specific survival analysis of 703 primary melanomas and (B) Kaplan–Meier overall survival analysis of 360 TCGA metastatic melanomas stratified by median *XAF1* RNA expression. Low is defined as bellow median. Analyses performed applying univariate Cox proportional hazard model. (C) Association between *XAF1* and *MX2* RNA expression tested by Pearson's correlation in primary melanomas from the LMC and (D) in metastatic melanomas from the TCGA. (E) The violin plots show the expression levels (log2(TPM +1)) of each *XAF1* isoform in the TCGA skin cutaneous melanoma (SKCM) samples. Graph created using online tool Gepia2.^38^ (F) Survival heatmap based on expression levels of different *XAF1* isoforms in several cancer types. Heatmap displays hazard ratios in logarithmic scale (log10); red blocks denote higher risks, and blue blocks denote lower risks. Framed blocks represent significant results in prognostic analyses. Heatmap created with Gepia2 ^38^ online tool

Since *MX2* expression has been associated with better MSS, and MX2 effects are partially mediated by XAF1, we examined relationship of *XAF1* mRNA with MSS using primary tumors of the Leeds Melanoma Cohort (LMC).^28^, ^37^ As seen in Figure 5A, higher *XAF1* expression was significantly associated with a longer MSS (HR =0.57, *p* < 0.001). A similar observation was made using TCGA metastatic melanomas testing overall survival (HR =0.57, *p* < 0.001, Figure 5B).

Furthermore, we analyzed the relationship between *XAF1* expression and clinicopathological parameters in the LMC. A significant negative correlation was observed between *XAF1* expression and Breslow thickness (*R* = −0.21, *p* < 0.001) (Figure [Link], [Link]). In both cohorts, *XAF1* and *MX2* RNA expressions were strongly correlated (*R* = 0.54 in LMC and *R* = 0.34 in TCGA) (Figure 5C,D).

*XAF1* exists in several isoforms; however, their function and prognostic significance in cancer remain an open question. We first checked the distribution of *XAF1* isoforms in the TCGA melanoma samples using the GEPIA2 analysis tool.^38^ As seen in violin plots of Figure 5E, melanoma tumors most abundantly expressed the full‐length *XAF1* transcript, while several other short truncated transcripts were also detected. Next, we tested the prognostic impact of these isoforms on the overall patient survival in several cancer types from the TCGA. The survival heatmap (Figure 5F) displays hazard ratios for different *XAF1* isoforms, where transcript expression was stratified by the median. Interestingly, expression of some *XAF1* transcripts was a negative prognostic factor in several cancer types, such as kidney renal clear cell carcinoma (KIRC), low‐grade glioma (LGG), and pancreatic adenocarcinoma (PAAD), while in melanoma (SKCM), all the transcripts were associated with a favorable outcome. Our immunoblotting data of melanoma cell lines (Figures 3B and 4A) show that the XAF1 antibody detects two separate bands with both being downregulated after the addition of the *XAF1* siRNA, suggesting that different *XAF1* isoforms may be present in these cell lines. Furthermore, MX2 downregulation resulted in a reduction of both bands, indicating that MX2 is involved in the regulation of both. Collectively, our data suggest that XAF1 might be partially mediating MX2 growth effects in melanoma tumors as well.

### 3.6 MX2 overexpression sensitizes melanoma cells to MAPK pathway targeted therapy

While MX2 overexpression renders melanoma cells less proliferative,^5^ previously, it has been shown that activation of the IFN pathway and higher XAF1 expression are associated with increased cell sensitivity to cytotoxic treatments.^16^, ^39^ Therefore, we evaluated the role of MX2 and XAF1 overexpression in targeted therapy response by incubating *BRAF* mutant WM983b^MX2high^ cells with 2‐µM vemurafenib and *NRAS* mutant WM1366^MX2high^ cells with 100‐nM trametinib for 72 h, before assessing cell viability.

**FIGURE 6:**
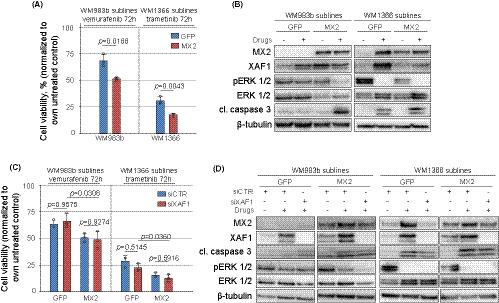
MX2 sensitizes melanoma cells to targeted therapy. (A) GFP‐ and MX2‐overexpressing WM983b melanoma cells were treated with 2‐µM vemurafenib and WM1366—with 100‐nM trametinib for 72 h. Cell viability was evaluated using CellTiter‐Glo luminescent viability assay. Luminescent signal of treated cells was normalized to respective untreated controls. Each circle on histogram depicts a mean value of an independent experiment and each bar represents the mean + SD (*n* = 3). Statistical significance was evaluated using Welch's *t* test after log transformation of the data. (B) Cells were treated as in (A) and protein expression analysis by immunoblotting was performed to evaluate MAPK pathway activity and apoptosis. (C) GFP/MX2‐overexpressing melanoma cells were transfected with *XAF1* targeting siRNA or scrambled control (siCTR) 24 h prior to 2‐µM vemurafenib treatment of WM983b sublines and 100‐nM trametinib treatment of WM1366 sublines. After 72 h, CellTiter‐Glo luminescent viability assay performed to assess cell viability and (D) immunoblotting analysis for protein expression. Each circle on histogram depicts mean value of an independent experiment and each bar represents the mean + SD (*n* = 3). Statistical significance was assessed by one‐way ANOVA with Tukey's multiple comparison test after log transformation of the data

We observed that MX2‐overexpressing cells were more sensitive to vemurafenib and trametinib than respective GFP controls (Figure 6A) which was accompanied by a higher caspase 3 cleavage (Figure 6B).

Interestingly, inhibition of the ERK pathway resulted in the upregulation of MX2 expression in GFP control lines although this was more pronounced in WM1366 cells. XAF1 upregulation was observed in both lines. Furthermore, in WM983b MX2‐overexpressing cells, vemurafenib treatment led to more potent pERK inhibition than seen in the GFP control cells (Figure 6B).

To check if the increased cell sensitivity of MX2‐overexpressing sublines was due to increased levels of XAF1, we knocked down XAF1 prior to the respective drug treatments. As seen in Figure 6C, XAF1 knockdown did not result in a reversal of sensitivity or decrease in caspase cleavage (Figure 6D), and MX2‐overexpressing sublines remained more vulnerable to targeted therapy compared to respective GFP controls, suggesting that MX2 sensitizes cells to MAPK inhibition independently of XAF1.

## 4 DISCUSSION

In this study, we aimed to elucidate the molecular mechanisms and networks affected by the antiviral *MX2* gene, previously reported to have tumor‐suppressive features in melanoma partially through negative modification of AKT activity, cell cycle, and tumor growth. Furthermore, MX2 is found downregulated during disease progression and associated with melanoma‐specific patient survival.^5^

*MX2* is an IFN response gene, and IFN signaling is frequently downregulated in melanoma ^6^ which could explain previously observed reduction of MX2 in melanoma samples. IFN signaling can exert both tumor‐promoting and tumor‐suppressive functions in various cancer types including melanoma.^40^, ^41^, ^42^ Interestingly, here, we show that MX2 overexpression contributes to the establishment of an IFN signature in melanoma cells and that MX2 is involved in IFN regulatory networks. In accordance with our results, a recent study reported that MX2 is expressed at relatively high levels in normal human melanocytes even without IFN stimulation and its expression was associated with immune‐response genes.^43^

Considering that IFN signaling defects are shown to contribute to immunotherapy resistance in melanoma patients,^44^ re‐establishing and enhancing the IFN signature in tumor cells via reactivation of regulators like MX2 might improve clinical responses.

Our data show that MX2 is involved in regulation of the STAT1 phosphorylation and activation. STAT1 activation is a highly complex process,^45^, ^46^ but upon type I IFN stimulation, it is canonically performed by JAK1 and TYK2 kinases in association with IFNAR transmembrane receptor cytoplasmic domain. Until now, no kinase activity has been attributed to MX2. It is unlikely that MX2 is directly involved in STAT1 phosphorylation. However, it cannot be excluded that MX2 is facilitating interactions with other proteins necessary for the phosphorylation.

In this study, we also show that MX2 can regulate XAF1. Our RNA‐seq analysis revealed *XAF1* as one of the top candidate genes induced by MX2 overexpression, which was also observed in several other melanoma cell lines and primary human melanocytes engineered to overexpress MX2. siRNA‐mediated knockdown of MX2 resulted in the downregulation of XAF1 regardless of endogenous levels or activation status of STAT1, suggesting that MX2 is necessary for full XAF1 induction by IFN. It has been previously reported that XAF1 induction is blocked by IRF1 depletion.^33^ We also show that IRF1 expression is dependent on MX2 status, which further supports the hypothesis of MX2 acting as an important regulatory node in the IFN signaling network.

We demonstrated that XAF1‐depleted cells become more proliferative possibly due to increased activation of the AKT pathway, an effect that is cell type and context dependent. This is in accordance with one previous study, showing that XAF1 could inhibit AKT signaling in gastric cancer cells.^47^ Moreover, we have previously demonstrated that MX2 overexpression suppresses growth of melanoma cells and tumors partially by reducing the AKT pathway activity ^5^ and results presented here suggest that XAF1 contributes to this regulation. Evidence of complex crosstalk between the PI3K/AKT and IFN signaling in melanoma and other cells has been established before.^48^, ^49^, ^50^ We saw that XAF1 knockdown increased proliferation of *NRAS*, but not *BRAF* mutant melanoma cells, yet we acknowledge that this effect was observed in a limited number of cell lines. Previous study has reported that mutations and deletions of *PTEN*, negative AKT activation regulator, are more frequent in *BRAF* mutant rather than *NRAS* mutant melanomas.^51^ To the best of our knowledge, *XAF1* and *PTEN* relationship in melanoma has not been studied; nevertheless, strongly reduced XAF1 levels were detected in *PTEN*‐null mouse prostate tumors,^52^ suggesting that regulatory mechanism between these two genes may exist.

We detected relatively low *XAF1* RNA levels in normal human melanocytes, while they were significantly higher in two primary and four metastatic melanoma lines. This observation may seem somewhat contradictory to a report by Ng et al.,^17^ where the authors observed significantly reduced XAF1 protein expression in primary melanomas compared to benign melanocytic nevi. However, in benign nevi, which are considered senescent lesions, XAF1 might be necessary for senescence regulation as previously reported.^53^

Our transcriptomic data analysis showed that high *XAF1* mRNA expression both in primary and metastatic tumors was associated with better patient survival. We observed a strong correlation between *MX2* and *XAF1* in the primary melanomas, while in metastatic tumors from this correlation was weaker, although still significant. This might suggest that during the disease progression regulation mechanism between *MX2* and *XAF1* are influenced by other factors.

It has been demonstrated that in prostate cancer full‐length XAF1 might be downregulated while truncated isoforms are expressed.^54^ However, such tendency was not observed in colorectal cancer where downregulation of full‐length XAF1 was accompanied with reduced expression of other variants as well.^16^ Furthermore, the same authors demonstrated that all *XAF1* transcripts were able to inhibit cell growth when overexpressed, although at different efficacy. In the TCGA melanoma tumors, we observed a predominant full‐length *XAF1* expression, and presence of all transcripts was associated with better overall survival. This was not the case for several other cancer types, highlighting *XAF1* role in a tissue‐specific manner.

Interestingly, we showed that in melanoma cells, MX2 mediates growth‐inhibitory effects of IFNα in a highly cell‐specific manner either via XAF1 or XAF1 independently. We did not observe any correlation with *BRAF*/*NRAS* status as before, suggesting that these differences might be influenced by other ISGs downstream of *MX2* and *XAF1* and/or additional genetic mutations that are important in the context of IFNα treatment. Several reports highlight complex interactions between XAF1 and p53 and suggest that XAF1 effects may partially be mediated via p53.^12^, ^55^, ^56^ Also, we observed that in WM983b and WM852 cells that lack functional p53, XAF1 had little effect on cell growth while in WM1366 cells with functional p53 pathway, XAF1 downregulation could reverse growth‐inhibitory effects of IFN and MX2. In support of our results, similar observations were made in HCT116 *TP53* wild‐type cells where ectopic expression of XAF1 markedly repressed xenograft tumor growth. In contrast, genetically engineered HCT116 cells that lack endogenous p53 (*TP53*−/−) were resistant to XAF1 overexpression and continued to grow in vivo.^12^ MX2 and p53 relationship has not been studied extensively; however, Forys et al. showed that loss of both p53 and ARF induces expression of ISGs, including *MX2*,^57^ and we can speculate that this may be a feedback to balance the absence of negative cell cycle regulators.

Interestingly, it should be noted that MX2 overexpression also led to a significant decrease of *DSG2* expression which has previously been associated with unfavorable melanoma outcome.^58^ Jointly, these results provide further explanation why MX2 expression is a predictor of better patient survival in melanoma.

In addition to mediating growth‐inhibitory effects of IFN, MX2 expression increased the sensitivity of melanoma cells to MAPK pathway inhibitors. Previously, it has been reported that in colon cancer, XAF1 expression is upregulated after inhibition of the MAPK pathway through transcriptional regulation, which mediated apoptosis.^35^ However, even though we did observe an upregulation of XAF1 following MAPK pathway inhibition in melanoma cells, XAF1 downregulation did not affect responses to the drugs indicating that MX2 sensitization is achieved independent of XAF1. It has also been shown that type I IFN treatment can enhance the cytotoxic effect of MEK inhibition in melanoma cell lines with low activity of IFN pathway ^39^ and since MX2 can induce IFN response profile affecting multiple genes, it is likely that these additional downstream factors are mediating this sensitization.

In summary, our study provides the first evidence of important and novel role of MX2 in IFN signaling network, where it is necessary for STAT1 phosphorylation. We also show that MX2 mediates IFN inhibitory effects in melanoma, sensitizes melanoma cells to MAPK pathway targeted therapy, and regulates XAF1 that has prognostic impact in melanoma patients. However, the findings presented in this study have to be seen in the light of certain limitations, which should be addressed in further studies. These include relatively few cell lines studied to draw firm conclusions on the genetic background contribution and lack of correct microenvironmental context, including immune cell components that influence outcome of IFN signaling.

## CONFLICT OF INTEREST

The authors declare no conflicts of interest.

## Supporting information

Figure S1

Figure S2

Figure S3

Figure S4

Figure S5

Figure S6

Figure S7

Figure S8

Figure S9

Figure S10

Figure S11

Table S1

Table S2

## Data Availability

Data supporting the findings of this study are available upon reasonable request from the corresponding author, A. S.

## References

[1] Amos CI , Wang L‐E , Lee JE , et al. Genome‐wide association study identifies novel loci predisposing to cutaneous melanoma†. Hum Mol Genet. 2011;20(24):5012‐5023. 21926416 10.1093/hmg/ddr415 PMC3298855

[2] Barrett JH , Iles MM , Harland M , et al. Genome‐wide association study identifies three new melanoma susceptibility loci. Nat Genet. 2011;43(11):1108‐1113. 21983787 10.1038/ng.959 PMC3251256

[3] Bishop DT , Demenais F , Iles MM , et al. Genome‐wide association study identifies three loci associated with melanoma risk. Nat Genet. 2009;41(8):920‐925. 19578364 10.1038/ng.411 PMC2741419

[4] Gibbs DC , Orlow I , Kanetsky PA , et al. Inherited genetic variants associated with occurrence of multiple primary melanoma. Cancer Epidemiol Biomark Prev. 2015;24(6):992. 10.1158/1055-9965.EPI-14-1426 PMC4452425 25837821

[5] Juraleviciute M , Pozniak J , Nsengimana J , et al. MX 2 is a novel regulator of cell cycle in melanoma cells. Pigment Cell & Melanoma Research. 2020;33(3):446‐457. 31660681 10.1111/pcmr.12837 PMC7180100

[6] Alavi S , Stewart AJ , Kefford RF , Lim SY , Shklovskaya E , Rizos H . Interferon signaling is frequently downregulated in melanoma. Front Immunol. 2018;9:1414. 29977240 10.3389/fimmu.2018.01414 PMC6021492

[7] Critchley‐Thorne RJ , Yan N , Nacu S , Weber J , Holmes SP . Lee pp. Down‐regulation of the interferon signaling pathway in T lymphocytes from patients with metastatic melanoma. PLoS Med. 2007;4(5):e176. 17488182 10.1371/journal.pmed.0040176 PMC1865558

[8] Zaretsky JM , Garcia‐Diaz A , Shin DS , et al. Mutations associated with acquired resistance to PD‐1 blockade in melanoma. N Engl J Med. 2016;375(9):819‐829. 27433843 10.1056/NEJMoa1604958 PMC5007206

[9] Gao J , Shi LZ , Zhao H , et al. Loss of IFN‐γ pathway genes in tumor cells as a mechanism of resistance to anti‐CTLA‐4 therapy. Cell. 2016;167(2):397‐404.e9. 27667683 10.1016/j.cell.2016.08.069 PMC5088716

[10] Wiesauer I , Gaumannmüller C , Steinparzer I , Strobl B , Kovarik P . Promoter occupancy of STAT1 in interferon responses is regulated by processive transcription. Mol Cell Biol. 2015;35(4):716. 25512607 10.1128/MCB.01097-14 PMC4301719

[11] Liston P , Fong WG , Kelly NL , et al. Identification of XAF1 as an antagonist of XIAP anti‐Caspase activity. Nat Cell Biol. 2001;3(2):128‐133. 11175744 10.1038/35055027

[12] Lee M‐G , Han J , Jeong S‐I , et al. XAF1 directs apoptotic switch of p53 signaling through activation of HIPK2 and ZNF313. Proc Natl Acad Sci. 2014;111(43):15532‐15537 25313037 10.1073/pnas.1411746111 PMC4217407

[13] Leaman DW , Chawla‐Sarkar M , Vyas K , et al. Identification of X‐linked inhibitor of apoptosis‐associated factor‐1 as an interferon‐stimulated gene that augments TRAIL Apo2L‐induced apoptosis. J Biol Chem. 2002;277(32):28504‐28511. 12029096 10.1074/jbc.M204851200

[14] Micali OC , Cheung HH , Plenchette S , et al. Silencing of the XAF1 gene by promoter hypermethylation in cancer cells and reactivation to TRAIL‐sensitization by IFN‐β. BMC Cancer. 2007;7(1):52. 17376236 10.1186/1471-2407-7-52 PMC1845166

[15] Lee M‐G , Huh J‐S , Chung S‐K , et al. Promoter CpG hypermethylation and downregulation of XAF1 expression in human urogenital malignancies: implication for attenuated p53 response to apoptotic stresses. Oncogene. 2006;25(42):5807‐5822. 16909101 10.1038/sj.onc.1209867

[16] Chung S , Lee M , Ryu B , et al. Frequent alteration of XAF1 in human colorectal cancers: implication for tumor cell resistance to apoptotic stresses. Gastroenterology. 2007;132(7):2459‐2477. 17570219 10.1053/j.gastro.2007.04.024

[17] Ng KCP , Campos EI , Martinka M , Li G . XAF1 expression is significantly reduced in human melanoma. Journal of Investigative Dermatology. 2004;123(6):1127‐11234. 10.1111/j.0022-202X.2004.23467.x 15610524

[18] Flørenes VA , Flem‐Karlsen K , McFadden E , et al. A three‐dimensional ex vivo viability assay reveals a strong correlation between response to targeted inhibitors and mutation status in melanoma lymph node metastases. Translational Oncology. 2019;12(7):951‐958. 31096111 10.1016/j.tranon.2019.04.001 PMC6520638

[19] Magnussen GI , Holm R , Emilsen E , Rosnes AKR , Slipicevic A , Flørenes VA . High expression of Wee1 is associated with poor disease‐free survival in malignant melanoma: potential for targeted therapy. PLoS One. 2012;7(6):e38254. 22719872 10.1371/journal.pone.0038254 PMC3373567

[20] Dobin A , Davis CA , Schlesinger F , et al. STAR: ultrafast universal RNA‐seq aligner. Bioinformatics. 2012;29(1):15‐21. 23104886 10.1093/bioinformatics/bts635 PMC3530905

[21] Liao Y , Smyth GK , Shi W . The R package Rsubread is easier, faster, cheaper and better for alignment and quantification of RNA sequencing reads. Nucleic Acids Res. 2019;47(8):e47. 30783653 10.1093/nar/gkz114 PMC6486549

[22] Love MI , Huber W , Anders S . Moderated estimation of fold change and dispersion for RNA‐seq data with DESeq2. Genome Biol. 2014;15(12):550. 25516281 10.1186/s13059-014-0550-8 PMC4302049

[23] Stephens M . False discovery rates: a new deal. Biostatistics. 2016;18(2):275‐294. 10.1093/biostatistics/kxw041 PMC5379932 27756721

[24] Eden E , Navon R , Steinfeld I , Lipson D , Yakhini Z . GOrilla: a tool for discovery and visualization of enriched GO terms in ranked gene lists. BMC Bioinformatics. 2009;10(1):48. 19192299 10.1186/1471-2105-10-48 PMC2644678

[25] Eden E , Lipson D , Yogev S , Yakhini Z . Discovering motifs in ranked lists of DNA sequences. PLoS Comput Biol. 2007;3(3):e39. 17381235 10.1371/journal.pcbi.0030039 PMC1829477

[26] Team RC . R: a language and environment for statistical computing. Vienna, Austria; 2020. Available from: https://www.r‐project.org/.

[27] Wickham H . ggplot2: Elegant Graphics for Data Analysis, 2nd edn. Springer‐Verlag; 2016.

[28] Nsengimana J , Laye J , Filia A , et al. β‐Catenin–mediated immune evasion pathway frequently operates in primary cutaneous melanomas. J Clin Investig. 2018;128(5):2048‐2063. 29664013 10.1172/JCI95351 PMC5919828

[29] Sun Y , Qiao L , Xia H‐X , et al. Regulation of XAF1 expression in human colon cancer cell by interferon β: activation by the transcription regulator STAT1. Cancer Lett. 2008;260(1):62‐71. 18035482 10.1016/j.canlet.2007.10.014

[30] Dicks MDJ , Betancor G , Jimenez‐Guardeño JM , et al. Multiple components of the nuclear pore complex interact with the amino‐terminus of MX2 to facilitate HIV‐1 restriction. PLoS Pathog. 2018;14(11):e1007408. 30496303 10.1371/journal.ppat.1007408 PMC6264145

[31] Barretina J , Caponigro G , Stransky N , et al. The Cancer Cell Line Encyclopedia enables predictive modelling of anticancer drug sensitivity. Nature. 2012;483(7391):603‐607. 22460905 10.1038/nature11003 PMC3320027

[32] Ghandi M , Huang FW , Jané‐Valbuena J , et al. Next‐generation characterization of the Cancer Cell Line Encyclopedia. Nature. 2019;569(7757):503‐508. 31068700 10.1038/s41586-019-1186-3 PMC6697103

[33] Jeong S‐I , Kim J‐W , Ko K‐P , et al. XAF1 forms a positive feedback loop with IRF‐1 to drive apoptotic stress response and suppress tumorigenesis. Cell Death Dis. 2018;9(8):806. 30042418 10.1038/s41419-018-0867-4 PMC6057933

[34] Tu SP , Liston P , Cui JT , et al. Restoration of XAF1 expression induces apoptosis and inhibits tumor growth in gastric cancer. Int J Cancer. 2009;125(3):688‐697. 19358264 10.1002/ijc.24282

[35] Yu LF , Wang J , Zou B , et al. XAF1 mediates apoptosis through an extracellular signal‐regulated kinase pathway in colon cancer. Cancer. 2007;109(10):1996‐2003. 17385215 10.1002/cncr.22624

[36] Zhu LM , Shi DM , Dai Q , et al. Tumor suppressor XAF1 induces apoptosis, inhibits angiogenesis and inhibits tumor growth in hepatocellular carcinoma. Oncotarget. 2014;5(14):5403‐5415. 24980821 10.18632/oncotarget.2114 PMC4170645

[37] Thakur R , Laye JP , Lauss M , et al. Transcriptomic analysis reveals prognostic molecular signatures of stage I melanoma. Clin Cancer Res. 2019;25(24):7424‐7435. 31515461 10.1158/1078-0432.CCR-18-3659 PMC7617074

[38] Tang Z , Kang B , Li C , Chen T , Zhang Z . GEPIA2: an enhanced web server for large‐scale expression profiling and interactive analysis. Nucleic Acids Res. 2019;47(W1):W556‐W560. 31114875 10.1093/nar/gkz430 PMC6602440

[39] Litvin O , Schwartz S , Wan Z , et al. Interferon α/β enhances the cytotoxic response of MEK inhibition in melanoma. Mol Cell. 2015;57(5):784‐796. 25684207 10.1016/j.molcel.2014.12.030 PMC4355234

[40] Budhwani M , Mazzieri R , Dolcetti R . Plasticity of type I interferon‐mediated responses in cancer therapy: from anti‐tumor immunity to resistance. Frontiers in Oncology. 2018;8:322. 30186768 10.3389/fonc.2018.00322 PMC6110817

[41] Mojic M , Takeda K , Hayakawa Y . The dark side of IFN‐γ: its role in promoting cancer immunoevasion. Int J Mol Sci. 2018;19(1):89 10.3390/ijms19010089 PMC5796039 29283429

[42] Jacquelot N , Yamazaki T , Roberti MP , et al. Sustained type I interferon signaling as a mechanism of resistance to PD‐1 blockade. Cell Res. 2019;29(10):846‐861. 31481761 10.1038/s41422-019-0224-x PMC6796942

[43] Choi J , Zhang T , Vu A , et al. Massively parallel reporter assays of melanoma risk variants identify MX2 as a gene promoting melanoma. Nat Commun. 2020;11(1):2718. 32483191 10.1038/s41467-020-16590-1 PMC7264232

[44] Manguso RT , Pope HW , Zimmer MD , et al. In vivo CRISPR screening identifies Ptpn2 as a cancer immunotherapy target. Nature. 2017;547(7664):413‐418. 28723893 10.1038/nature23270 PMC5924693

[45] Michalska A , Blaszczyk K , Wesoly J , Bluyssen HAR . A positive feedback amplifier circuit that regulates interferon (IFN)‐stimulated gene expression and controls type I and type II IFN responses. Front Immunol. 2018;9:1135. 29892288 10.3389/fimmu.2018.01135 PMC5985295

[46] Majoros A , Platanitis E , Kernbauer‐Hölzl E , Rosebrock F , Müller M , Decker T . Canonical and non‐canonical aspects of JAK–STAT signaling: lessons from interferons for cytokine responses. Front Immunol. 2017;8:29. 28184222 10.3389/fimmu.2017.00029 PMC5266721

[47] Sun PH , Zhu LM , Qiao MM , et al. The XAF1 tumor suppressor induces autophagic cell death via upregulation of Beclin‐1 and inhibition of Akt pathway. Cancer Lett. 2011;310(2):170‐180. 21788101 10.1016/j.canlet.2011.06.037

[48] Nguyen H , Ramana CV , Bayes J , Stark GR . Roles of phosphatidylinositol 3‐kinase in interferon‐γ‐dependent phosphorylation of STAT1 on serine 727 and activation of gene expression. J Biol Chem. 2001;276(36):33361‐33368. 11438544 10.1074/jbc.M105070200

[49] Kaur S , Sassano A , Dolniak B , et al. Role of the Akt pathway in mRNA translation of interferon‐stimulated genes. Proc Natl Acad Sci. 2008;105(12):4808‐4813. 18339807 10.1073/pnas.0710907105 PMC2290753

[50] Garcia‐Diaz A , Shin DS , Moreno BH , et al. Interferon receptor signaling pathways regulating PD‐L1 and PD‐L2 expression. Cell Rep. 2017;19(6):1189‐1201. 28494868 10.1016/j.celrep.2017.04.031 PMC6420824

[51] Akbani R , Akdemir K , Aksoy B , et al. Genomic classification of cutaneous melanoma. Cell. 2015;161(7):1681‐1696. 26091043 10.1016/j.cell.2015.05.044 PMC4580370

[52] Lunardi A , Ala U , Epping MT , et al. A co‐clinical approach identifies mechanisms and potential therapies for androgen deprivation resistance in prostate cancer. Nat Genet. 2013;45(7):747‐755. 23727860 10.1038/ng.2650 PMC3787876

[53] Heo J‐I , Kim W , Choi KJ , Bae S , Jeong J‐H , Kim KS . XIAP‐associating factor 1, a transcriptional target of BRD7, contributes to endothelial cell senescence. Oncotarget. 2016;7(5):5118‐5130. 26802028 10.18632/oncotarget.6962 PMC4868675

[54] Fang X , Liu Z , Fan Y , et al. Switch to full‐length of XAF1 mRNA expression in prostate cancer cells by the DNA methylation inhibitor. Int J Cancer. 2006;118(10):2485‐2489. 16353137 10.1002/ijc.21636

[55] Zou B , Chim CS , Pang R , et al. XIAP‐associated factor 1 (XAF1), a novel target of p53, enhances p53‐mediated apoptosis via post‐translational modification. Mol Carcinog. 2012;51(5):422‐432. 21678496 10.1002/mc.20807

[56] Schluckebier L , Aran V , De Moraes J , Paiva H , Sternberg C , Ferreira C G . XAF1 expression levels in a non‐small cell lung cancer cohort and its potential association with carcinogenesis. Oncol Rep. 2017;38:402‐410. 28560416 10.3892/or.2017.5680

[57] Forys Jason T , Kuzmicki Catherine E , Saporita Anthony J , Winkeler Crystal L , Maggi Leonard B , Weber JD . ARF and p53 coordinate tumor suppression of an oncogenic IFN‐β‐STAT1‐ISG15 signaling axis. Cell Rep. 2014;7(2):514‐526. 24726362 10.1016/j.celrep.2014.03.026 PMC4157460

[58] Tan LY , Mintoff C , Johan ZM , et al. Desmoglein 2 promotes vasculogenic mimicry in melanoma and is associated with poor clinical outcome. Oncotarget. 2016;7(29):46492‐46508. 27340778 10.18632/oncotarget.10216 PMC5216812

