# Supplementary figures and images for: MX2 mediates establishment of interferon response profile, regulates XAF1, and can sensitize melanoma cells to targeted therapy
Source: Cancer Med. 2021 Mar 18;10(8):2840–54. doi: 10.1002/cam4.3846 (PMC8026919; doi:10.1002/cam4.3846)

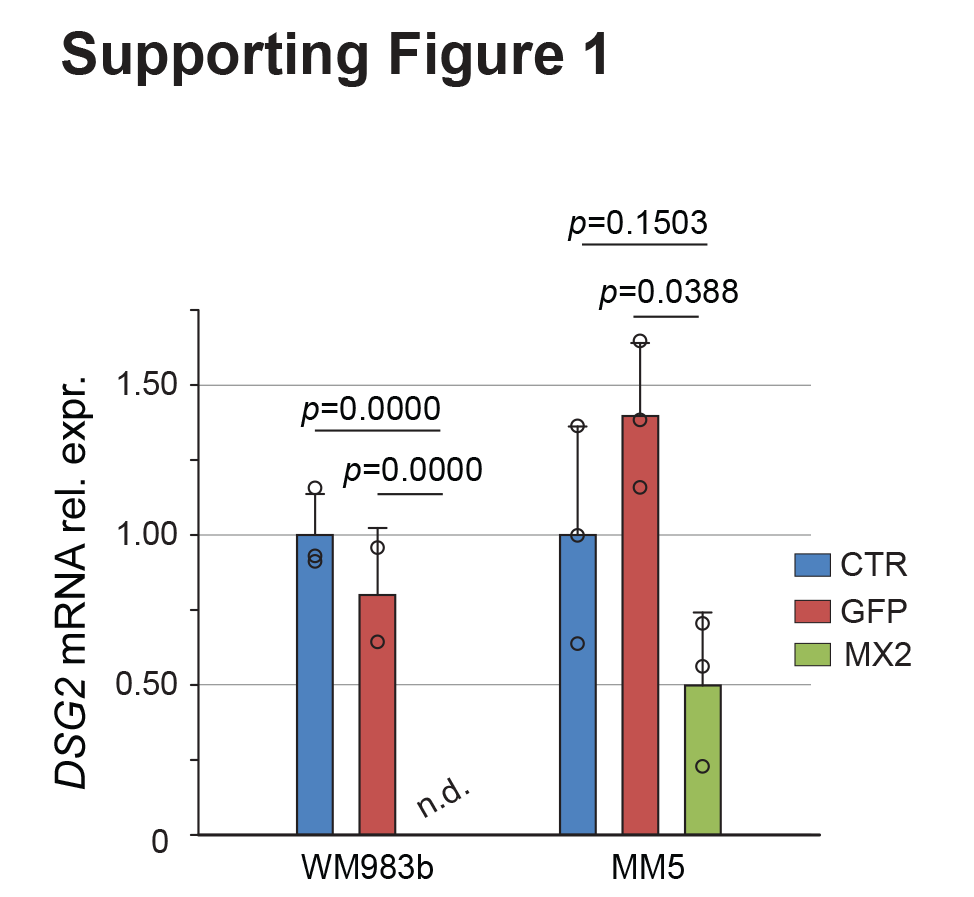

Supplement: Supplementary file 1 — Figure S1 [file CAM4-10-2840-s005.tif]

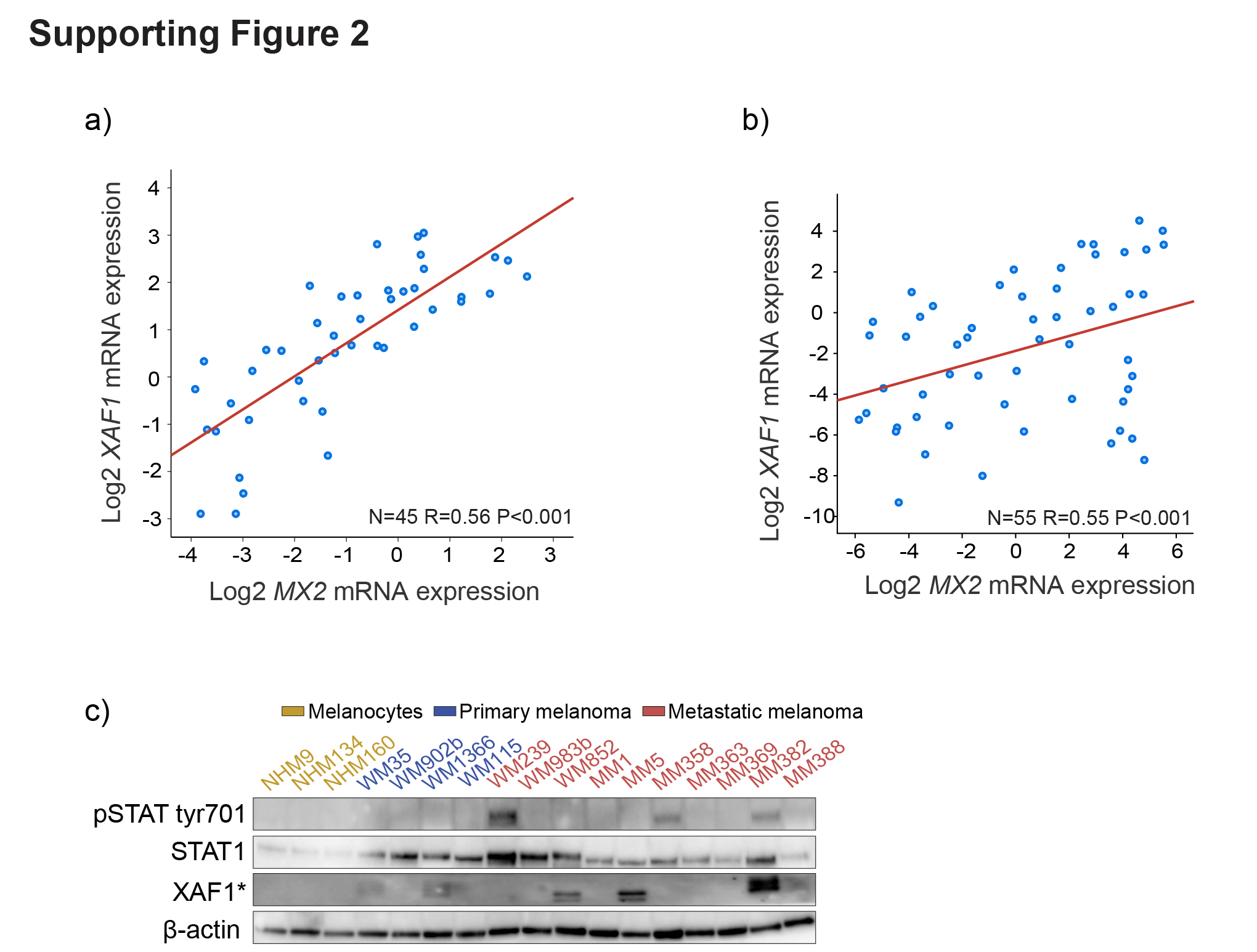

Supplement: Supplementary file 2 — Figure S2 [file CAM4-10-2840-s008.tif]

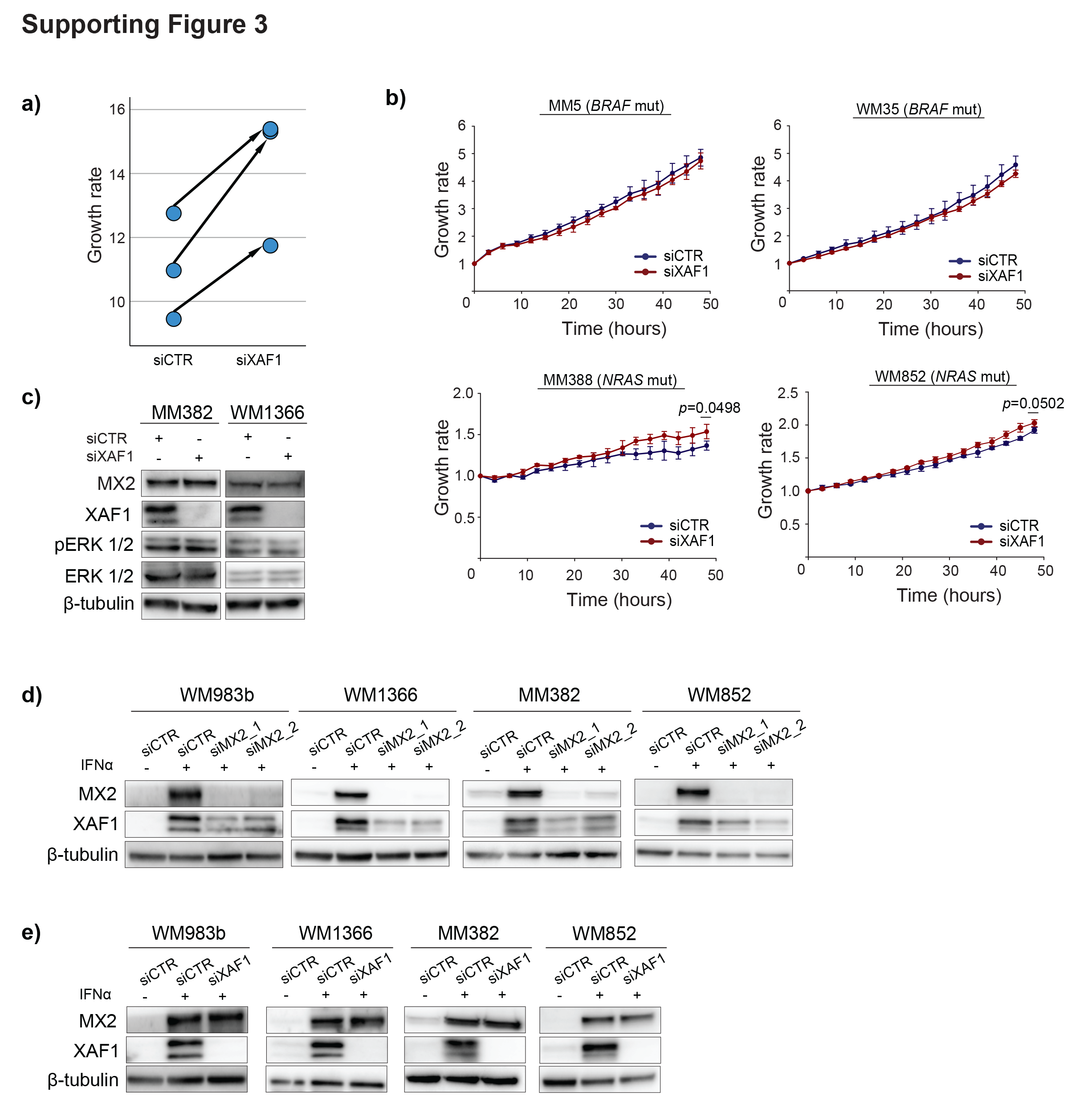

Supplement: Supplementary file 3 — Figure S3 [file CAM4-10-2840-s003.tif]

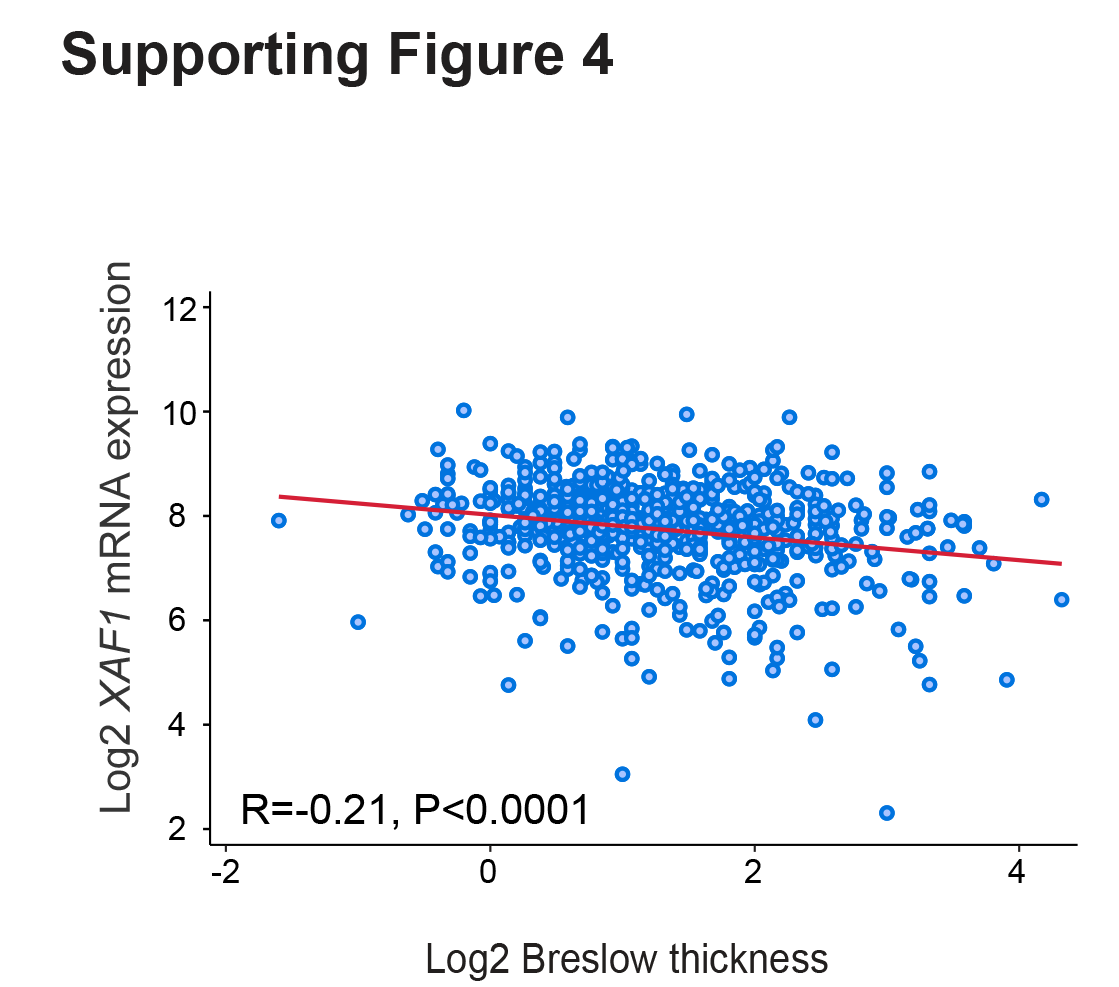

Supplement: Supplementary file 4 — Figure S4 [file CAM4-10-2840-s010.tif]

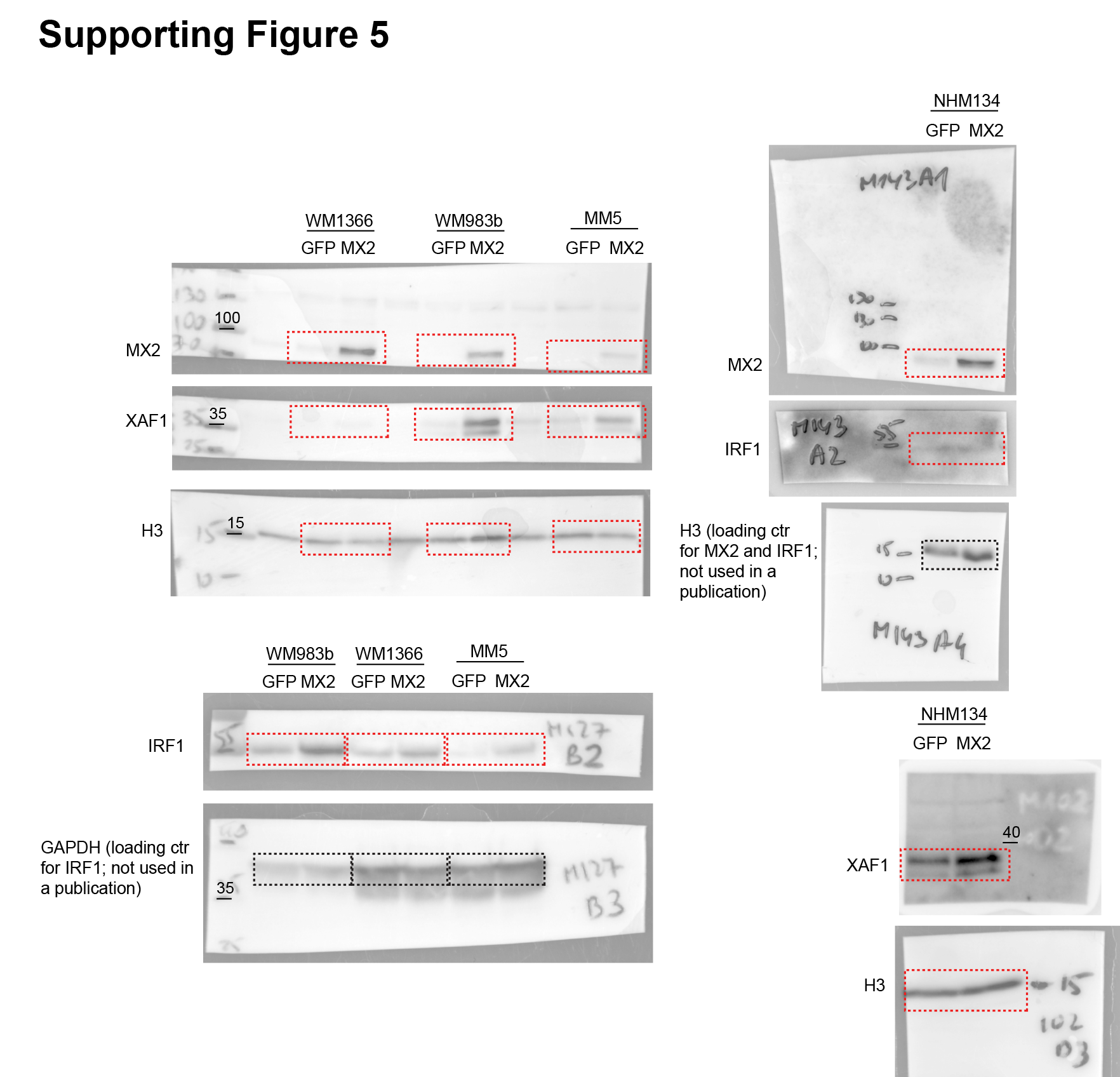

Supplement: Supplementary file 5 — Figure S5 [file CAM4-10-2840-s001.tif]

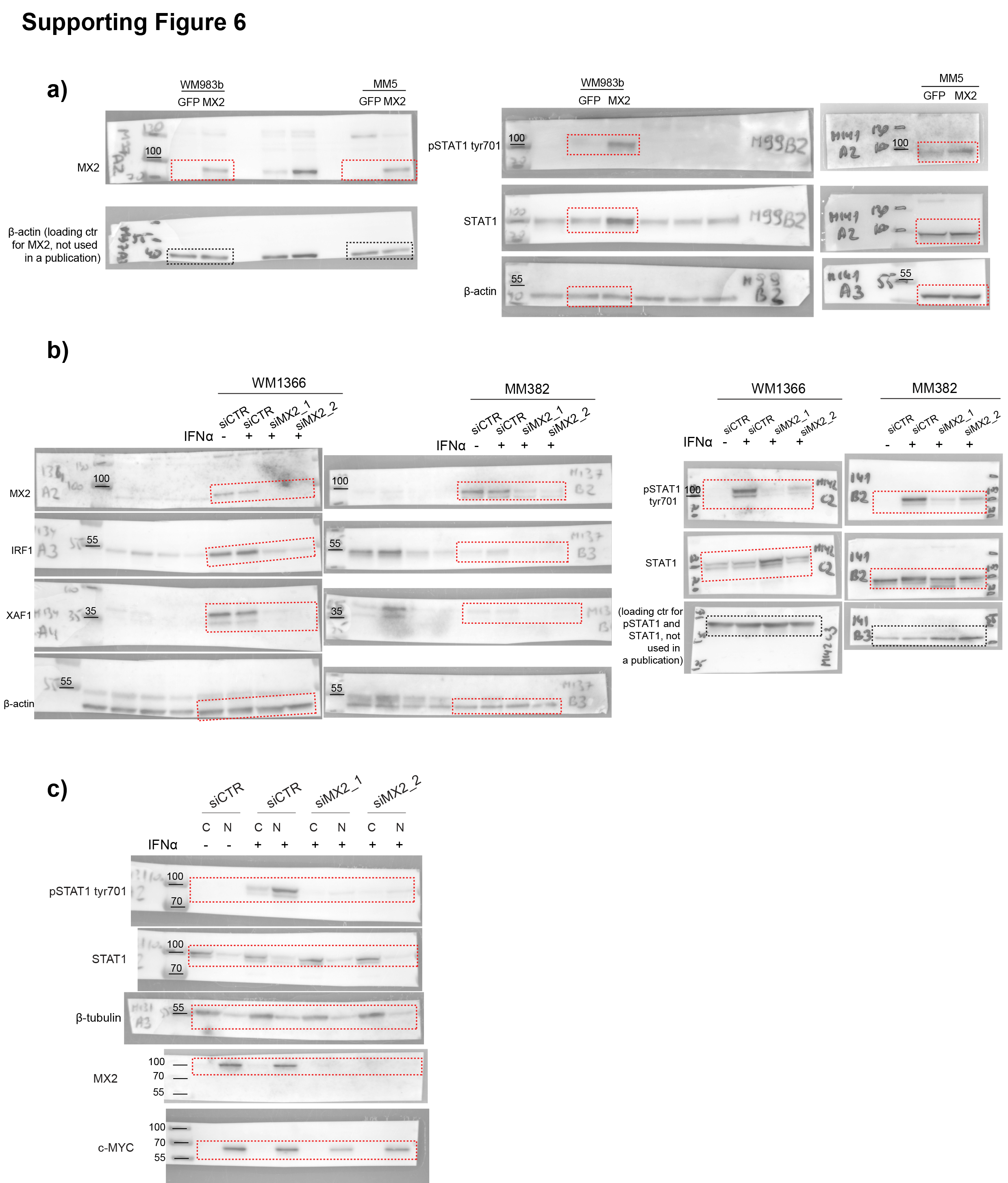

Supplement: Supplementary file 6 — Figure S6 [file CAM4-10-2840-s011.tif]

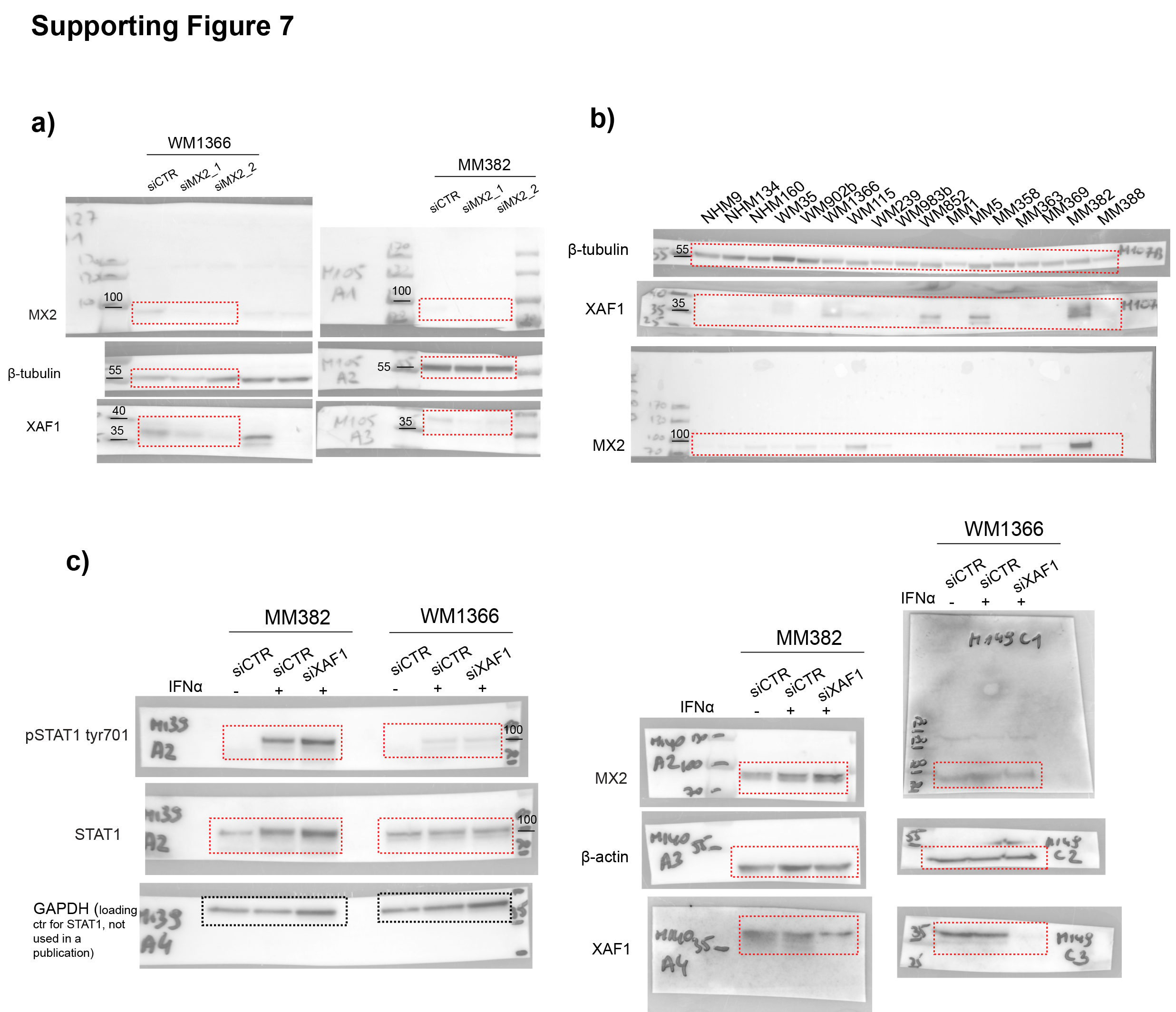

Supplement: Supplementary file 7 — Figure S7 [file CAM4-10-2840-s013.tif]

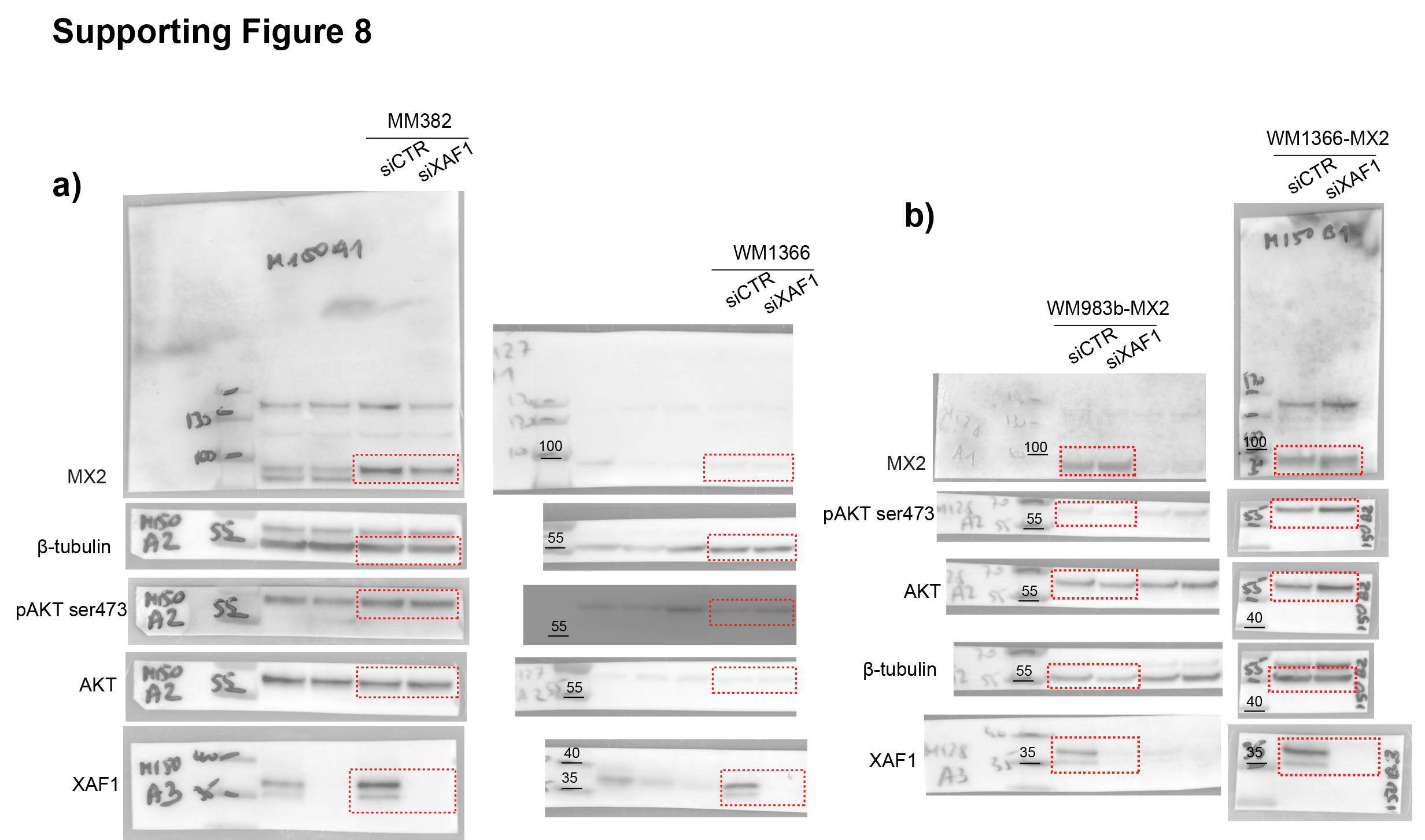

Supplement: Supplementary file 8 — Figure S8 [file CAM4-10-2840-s014.tif]

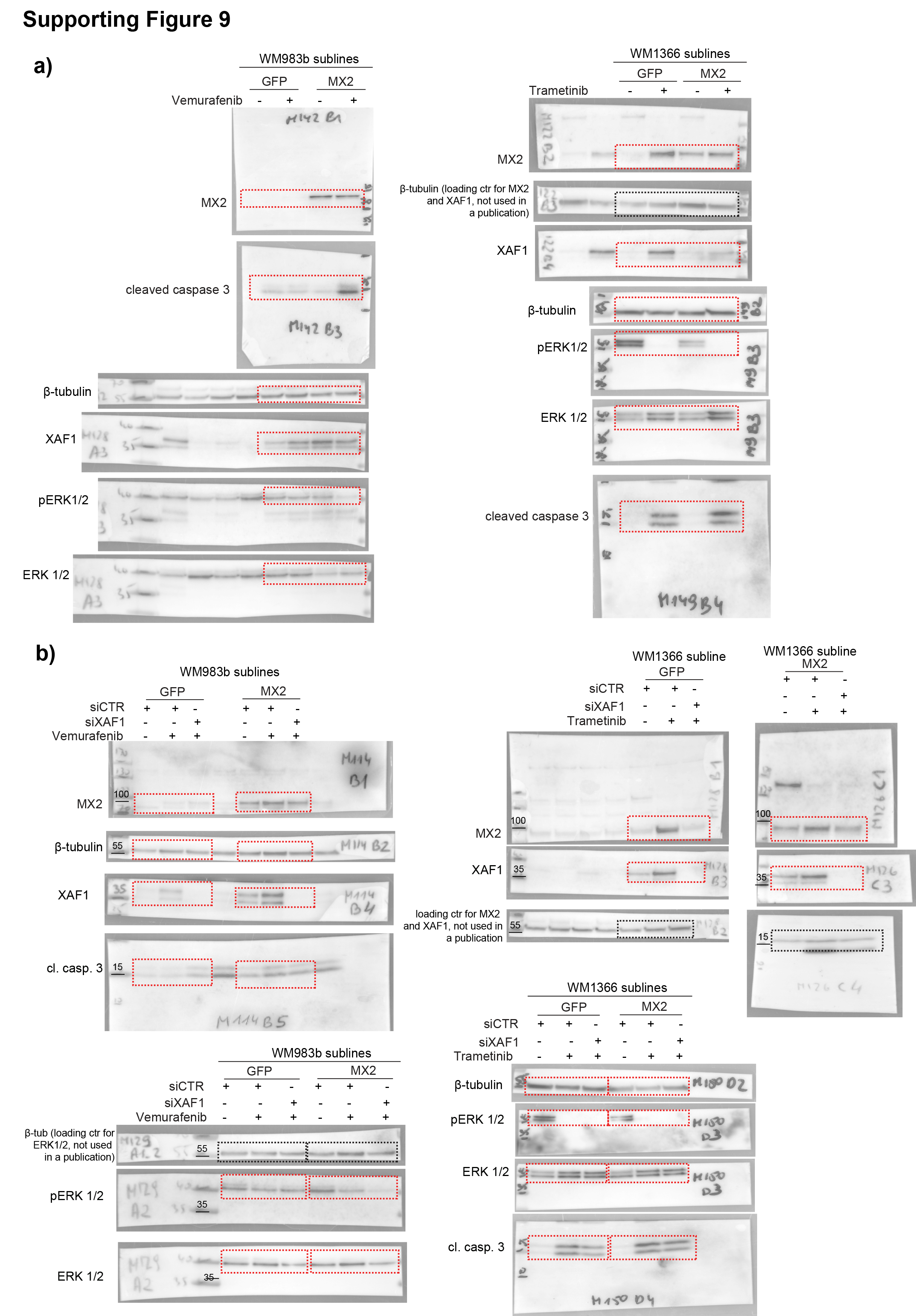

Supplement: Supplementary file 9 — Figure S9 [file CAM4-10-2840-s012.tif]

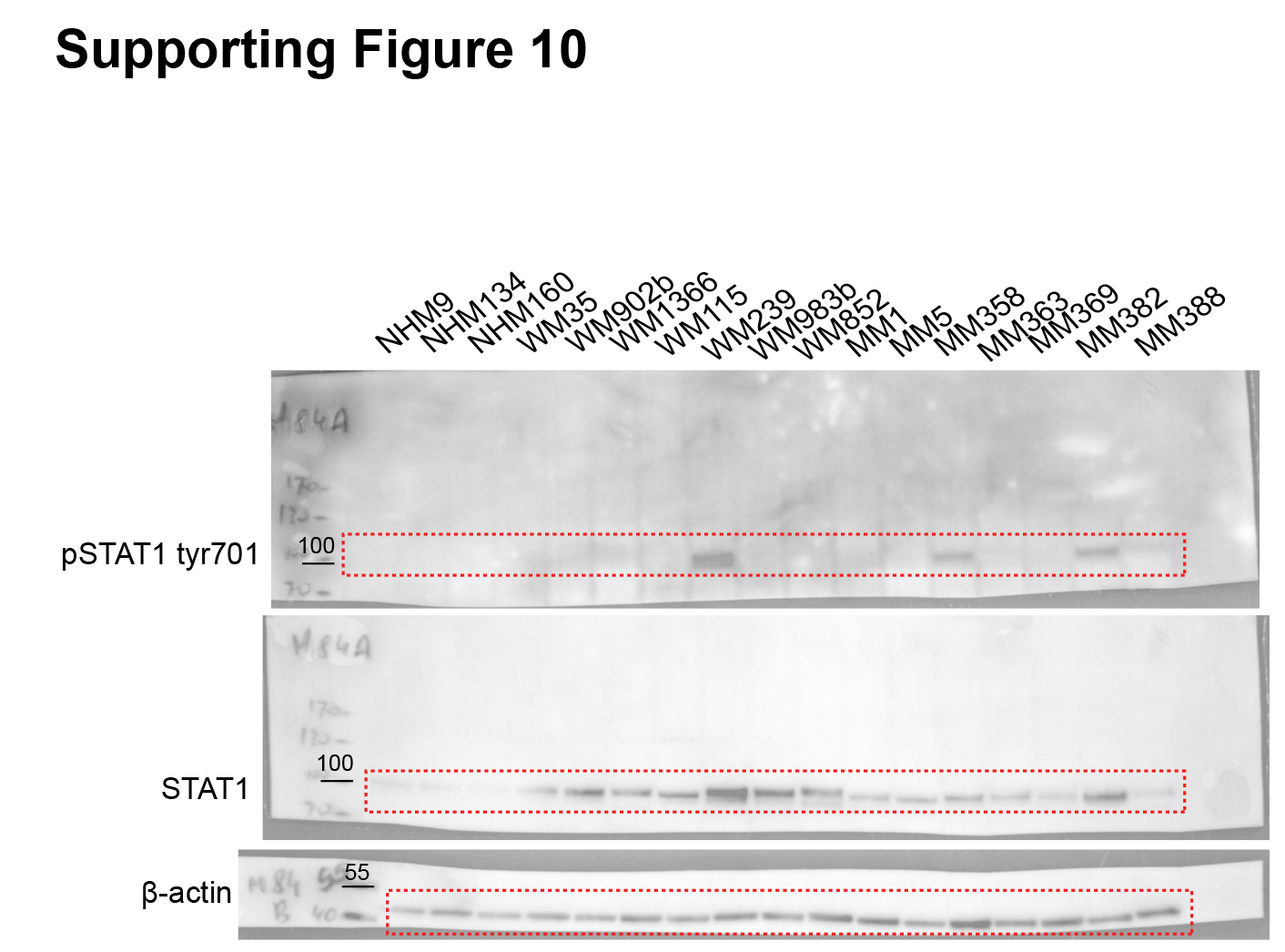

Supplement: Supplementary file 10 — Figure S10 [file CAM4-10-2840-s007.tif]

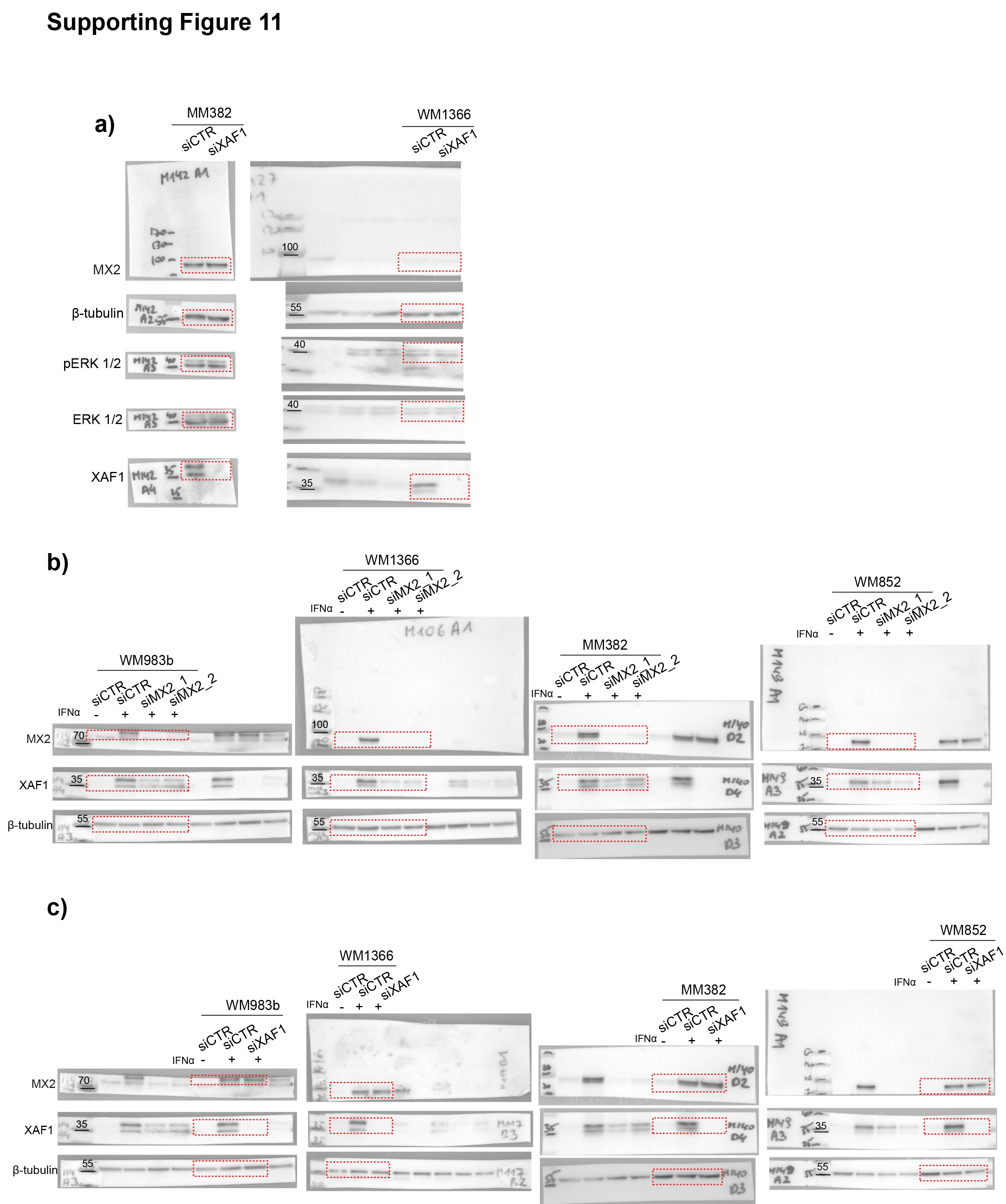

Supplement: Supplementary file 11 — Figure S11 [file CAM4-10-2840-s002.tif]
