# Supplementary material for: MX2 mediates establishment of interferon response profile, regulates XAF1, and can sensitize melanoma cells to targeted therapy
Source: Cancer Med. 2021 Mar 18;10(8):2840–54. doi: 10.1002/cam4.3846 (PMC8026919; doi:10.1002/cam4.3846)
Supplement: Supplementary file 12 — Table S1 [file CAM4-10-2840-s004.pdf]

Supporting Table 1. Commercial melanoma cell lines used in a study		
Cell line	Catalog number	Company
WM902b	WM902B-01-0001	Rockland Immunochemicals, Inc.
WM1366	WM1366-01-0001	Rockland Immunochemicals, Inc.
WM115	WM115-01-0001	Rockland Immunochemicals, Inc.
WM239	WM239A-01-0001	Rockland Immunochemicals, Inc.
WM983b	WM983B-01-0001	Rockland Immunochemicals, Inc.
WM852	WM852-01-0001	Rockland Immunochemicals, Inc.
