# Supplementary material for: MX2 mediates establishment of interferon response profile, regulates XAF1, and can sensitize melanoma cells to targeted therapy
Source: Cancer Med. 2021 Mar 18;10(8):2840–54. doi: 10.1002/cam4.3846 (PMC8026919; doi:10.1002/cam4.3846)
Supplement: Supplementary file 13 — Table S2 [file CAM4-10-2840-s009.pdf]

Supporting Table 2. Differentially expressed genes between WM983b MX2-overexpressing (MX2) and both GFP-overexpressing (GFP) and untransduced control (CTR)

geneID	Gene Symbol	log2(FoldChange) MX2 *vs* CTR	log2(FoldChange) MX2 *vs* GFP	P-value MX2 *vs* CTR	P-value MX2 *vs* GFP	Q-value MX2 *vs* CTR	Q-value MX2 *vs* GFP
ENSG00000165949	*IFI27*	7,090466	5,872172	1,81E-195	1,23E-87	1,84E-191	2,40E-84
ENSG00000171403	*KRT9*	6,72397	2,026172	2,06E-09	2,78E-06	3,77E-08	1,87E-05
ENSG00000196126	*HLA-DRB1*	6,177316	3,334438	3,81E-08	5,46E-07	5,65E-07	4,24E-06
ENSG00000108622	*ICAM2*	6,06522	1,612503	2,05E-08	2,30E-06	3,19E-07	1,57E-05
ENSG00000107165	*TYRP1*	5,969977	3,444863	0,00E+00	1,67E-17	0,00E+00	5,85E-16
ENSG00000163017	*ACTG2*	5,834478	2,603512	1,52E-47	1,63E-27	5,06E-45	1,73E-25
ENSG00000183486	*MX2*	5,601426	4,388222	8,25E-55	1,61E-17	3,90E-52	5,67E-16
ENSG00000251175	*GSTCD-AS1*	5,033454	4,29639	3,34E-06	1,91E-06	3,41E-05	1,33E-05
ENSG00000100368	*CSF2RB*	4,982238	2,662509	4,49E-14	3,72E-13	1,53E-12	7,60E-12
ENSG00000275385	*CCL18*	4,424097	3,346184	9,79E-18	1,21E-17	4,81E-16	4,33E-16
ENSG00000132530	*XAF1*	4,314567	1,373044	1,56E-85	1,05E-04	1,87E-82	5,13E-04
ENSG00000198502	*HLA-DRB5*	4,220669	3,318614	4,08E-06	7,53E-07	4,08E-05	5,69E-06
ENSG00000103184	*SEC14L5*	4,159618	1,986662	2,16E-14	9,61E-10	7,63E-13	1,18E-08
ENSG00000138944	*SHISAL1*	3,818538	2,863013	1,28E-09	5,55E-09	2,45E-08	6,03E-08
ENSG00000169313	*P2RY12*	3,675243	3,255046	7,84E-23	2,63E-26	5,99E-21	2,44E-24
ENSG00000162692	*VCAM1*	3,674105	3,388936	1,63E-06	4,00E-07	1,76E-05	3,20E-06
ENSG00000224940	*PRRT4*	3,574987	1,781419	5,61E-13	2,33E-08	1,71E-11	2,29E-07
ENSG00000142173	*COL6A2*	3,573173	-1,879486	5,21E-13	1,05E-20	1,59E-11	5,43E-19
ENSG00000172005	*MAL*	3,537834	3,051771	2,01E-19	3,75E-20	1,16E-17	1,79E-18
ENSG00000135914	*HTR2B*	3,500703	3,038299	5,68E-29	7,04E-29	6,74E-27	8,90E-27
ENSG00000135218	*CD36*	3,475321	1,882837	2,46E-26	2,12E-16	2,54E-24	6,55E-15
ENSG00000078900	*TP73*	3,456474	-1,99044	4,73E-15	3,07E-27	1,78E-13	3,18E-25
ENSG00000231389	*HLA-DPA1*	3,371145	3,431567	7,86E-48	1,11E-14	2,71E-45	2,81E-13
ENSG00000166780	*BMERB1*	3,293523	1,007519	1,86E-07	6,27E-04	2,43E-06	2,55E-03
ENSG00000185885	*IFITM1*	3,291265	1,471812	5,61E-11	1,61E-05	1,33E-09	9,37E-05
ENSG00000111335	*OAS2*	3,244288	2,690523	2,23E-84	3,50E-10	2,52E-81	4,60E-09
ENSG00000146242	*TPBG*	3,222586	1,92633	3,53E-35	2,86E-22	5,87E-33	1,77E-20
ENSG00000233098	*LOC339260*	3,132361	1,276036	1,55E-10	1,12E-05	3,45E-09	6,70E-05
ENSG00000268894	*PLCE1-AS1*	3,131466	2,505737	1,19E-06	1,65E-07	1,32E-05	1,41E-06
ENSG00000147257	*GPC3*	3,00585	2,494245	8,50E-05	4,07E-05	6,15E-04	2,17E-04
ENSG00000181634	*TNFSF15*	2,898998	1,889159	4,95E-76	1,75E-25	3,86E-73	1,50E-23
ENSG00000129595	*EPB41L4A*	2,841684	1,367877	9,98E-76	3,10E-31	7,23E-73	4,67E-29
ENSG00000204287	*HLA-DRA*	2,753845	3,51767	5,38E-13	1,11E-14	1,64E-11	2,82E-13
ENSG00000235770	*LINC00607*	2,649978	2,140572	1,39E-09	7,47E-09	2,64E-08	7,97E-08
ENSG00000132274	*TRIM22*	2,645769	2,357616	8,00E-09	7,06E-08	1,34E-07	6,40E-07
ENSG00000255494	*LINC00681*	2,607159	3,292159	1,66E-21	1,24E-36	1,13E-19	2,77E-34
ENSG00000205126	*ACCSL*	2,52045	2,183155	5,85E-10	3,71E-09	1,20E-08	4,15E-08
ENSG00000129521	*EGLN3*	2,422204	2,796176	5,10E-26	2,66E-49	5,10E-24	1,24E-46
ENSG00000251493	*FOXD1*	2,34343	-1,098635	2,70E-08	2,18E-07	4,09E-07	1,83E-06
ENSG00000168484	*SFTPC*	2,284673	2,152038	3,10E-07	1,34E-08	3,88E-06	1,37E-07
ENSG00000142156	*COL6A1*	2,280267	-1,281047	1,61E-32	3,59E-20	2,44E-30	1,72E-18
ENSG00000110318	*CEP126*	2,246996	1,264837	1,38E-13	5,32E-10	4,47E-12	6,77E-09
ENSG00000137959	*IFI44L*	2,200409	2,179875	1,39E-36	3,64E-06	2,58E-34	2,39E-05
ENSG00000126500	*FLRT1*	2,05996	-1,055179	9,74E-06	2,63E-05	8,89E-05	1,47E-04
ENSG00000121858	*TNFSF10*	1,997202	1,013505	7,04E-06	6,69E-04	6,66E-05	2,69E-03
ENSG00000214357	*NEURL1B*	1,993433	-1,072525	1,17E-05	4,56E-06	1,05E-04	2,94E-05
ENSG00000185046	*ANKS1B*	1,984259	1,397615	6,47E-32	3,23E-18	9,25E-30	1,24E-16
ENSG00000114757	*PEX5L*	1,947412	1,245441	9,46E-09	1,03E-05	1,56E-07	6,21E-05
ENSG00000137869	*CYP19A1*	1,940216	1,221553	1,75E-09	1,48E-06	3,28E-08	1,05E-05
ENSG00000108381	*ASPA*	1,935569	2,113297	7,23E-20	9,31E-32	4,36E-18	1,50E-29
ENSG00000113212	*PCDHB7*	1,912775	1,833326	8,58E-17	6,77E-18	3,87E-15	2,45E-16
ENSG00000133321	*PLAAT4*	1,866201	1,219319	1,05E-09	1,66E-08	2,03E-08	1,67E-07
ENSG00000188783	*PRELP*	1,857078	1,762981	2,05E-11	1,12E-17	5,14E-10	4,00E-16
ENSG00000155961	*RAB39B*	1,839691	2,022718	5,70E-10	9,90E-13	1,17E-08	1,93E-11
ENSG00000115474	*KCNJ13*	1,828847	3,131916	1,80E-14	9,24E-24	6,45E-13	6,64E-22
ENSG00000156127	*BATF*	1,818528	4,489294	6,25E-05	8,14E-07	4,71E-04	6,08E-06
ENSG00000169085	*VXN*	1,735127	1,377335	9,23E-10	1,79E-10	1,81E-08	2,47E-09
ENSG00000185585	*OLFML2A*	1,730166	-1,41938	3,90E-24	2,65E-49	3,29E-22	1,24E-46

ENSG00000226674	*TEX41*	1,729119	1,74071	7,95E-62	1,75E-67	4,48E-59	2,02E-64
ENSG00000154258	*ABCA9*	1,723683	1,315861	8,93E-42	3,02E-19	2,16E-39	1,29E-17
ENSG00000172348	*RCAN2*	1,710971	1,493086	7,28E-17	2,43E-15	3,30E-15	6,62E-14
ENSG00000091428	*RAPGEF4*	1,705406	2,76974	2,78E-15	1,81E-44	1,08E-13	6,10E-42
ENSG00000000971	*CFH*	1,677018	1,813102	8,87E-15	1,66E-22	3,26E-13	1,06E-20
ENSG00000206538	*VGLL3*	1,661413	-1,011411	1,64E-21	2,96E-11	1,12E-19	4,60E-10
ENSG00000089127	*OAS1*	1,601134	1,663478	9,02E-22	2,11E-21	6,34E-20	1,18E-19
ENSG00000145063	*FLJ33534*	1,578038	1,387109	5,76E-04	3,54E-04	3,28E-03	1,53E-03
ENSG00000144278	*GALNT13*	1,554053	1,723017	1,33E-10	6,15E-15	3,00E-09	1,61E-13
ENSG00000074590	*NUAK1*	1,530499	-1,143983	7,62E-06	5,67E-07	7,14E-05	4,37E-06
ENSG00000171561	*OR2AT4*	1,472967	1,042604	1,44E-04	4,25E-04	9,78E-04	1,80E-03
ENSG00000196576	*PLXNB2*	1,421215	-1,173377	3,09E-16	3,69E-29	1,33E-14	4,82E-27
ENSG00000142798	*HSPG2*	1,416711	-1,301635	9,72E-06	4,38E-38	8,88E-05	1,02E-35
ENSG00000146859	*TMEM140*	1,339684	2,09284	1,64E-30	2,52E-36	2,14E-28	5,55E-34
ENSG00000116574	*RHOU*	1,311866	-1,985165	1,31E-03	2,03E-10	6,71E-03	2,77E-09
ENSG00000118263	*KLF7*	1,288637	1,268916	2,15E-15	2,20E-15	8,44E-14	6,02E-14
ENSG00000182809	*CRIP2*	1,26461	-1,436068	2,63E-07	5,74E-18	3,36E-06	2,12E-16
ENSG00000137752	*CASP1*	1,259783	1,535697	2,03E-18	1,27E-38	1,07E-16	3,15E-36
ENSG00000177363	*LRRN4CL*	1,236058	2,370263	1,52E-05	3,88E-10	1,32E-04	5,07E-09
ENSG00000267339	*LINC00906*	1,225654	2,90837	2,30E-04	3,47E-08	1,47E-03	3,30E-07
ENSG00000150540	*HNMT*	1,217102	2,604649	1,75E-24	1,33E-85	1,56E-22	2,18E-82
ENSG00000087245	*MMP2*	1,184036	1,512181	1,27E-12	2,61E-18	3,73E-11	1,02E-16
ENSG00000116745	*RPE65*	1,181554	1,039768	1,82E-03	1,84E-03	8,91E-03	6,65E-03
ENSG00000162591	*MEGF6*	1,17254	1,020835	3,93E-10	5,07E-10	8,30E-09	6,47E-09
ENSG00000120328	*PCDHB12*	1,153928	1,113862	1,83E-11	2,95E-14	4,62E-10	7,08E-13
ENSG00000089041	*P2RX7*	1,149095	2,011669	2,77E-13	9,39E-46	8,73E-12	3,41E-43
ENSG00000134160	*TRPM1*	1,142456	2,173581	1,49E-04	4,27E-52	1,00E-03	2,33E-49
ENSG00000090975	*PITPNM2*	1,142447	-1,397186	6,04E-05	1,68E-13	4,57E-04	3,61E-12
ENSG00000107968	*MAP3K8*	1,126033	1,554947	6,74E-05	4,40E-07	5,03E-04	3,49E-06
ENSG00000154263	*ABCA10*	1,119662	2,018792	2,26E-07	2,87E-20	2,92E-06	1,42E-18
ENSG00000135205	*CCDC146*	1,103334	1,072544	5,54E-08	1,01E-08	7,99E-07	1,05E-07
ENSG00000170581	*STAT2*	1,095597	1,092503	1,54E-25	3,65E-32	1,48E-23	6,01E-30
ENSG00000142627	*EPHA2*	1,095321	-2,924565	3,18E-05	2,95E-63	2,58E-04	2,76E-60
ENSG00000136895	*GARNL3*	1,089848	1,19536	1,25E-06	3,99E-10	1,39E-05	5,20E-09
ENSG00000223865	*HLA-DPB1*	1,065771	3,057456	1,81E-03	4,88E-06	8,89E-03	3,12E-05
ENSG00000188958	*UTS2B*	1,050008	1,664305	5,07E-04	5,73E-06	2,95E-03	3,62E-05
ENSG00000168939	*SPRY3*	1,044985	1,109909	1,65E-08	3,32E-10	2,60E-07	4,39E-09
ENSG00000257704	*INAFM1*	1,006394	-1,418714	6,38E-04	4,20E-09	3,59E-03	4,65E-08
ENSG00000248964	*LOC101929470*	1,006051	2,353346	1,89E-03	5,99E-06	9,21E-03	3,77E-05
ENSG00000107821	*KAZALD1*	-1,038657	-1,405931	6,16E-05	1,12E-08	4,64E-04	1,16E-07
ENSG00000155034	*FBXL18*	-1,079928	-1,45079	1,62E-15	3,64E-35	6,44E-14	7,36E-33
ENSG00000146285	*SCML4*	-1,082111	2,134035	3,28E-11	1,82E-24	7,96E-10	1,40E-22
ENSG00000204934	*ATP6V0E2-AS1*	-1,084656	-1,012316	1,27E-05	1,01E-05	1,12E-04	6,10E-05
ENSG00000064989	*CALCRL*	-1,085246	1,267185	9,86E-06	5,40E-05	8,99E-05	2,82E-04
ENSG00000102313	*ITIH6*	-1,09764	-1,74258	1,52E-07	2,80E-13	2,01E-06	5,82E-12
ENSG00000171130	*ATP6V0E2*	-1,104169	-1,193557	2,19E-10	8,95E-16	4,77E-09	2,58E-14
ENSG00000168621	*GDNF*	-1,124226	-1,197857	6,17E-04	1,96E-04	3,49E-03	9,02E-04
ENSG00000143153	*ATP1B1*	-1,136165	-1,594021	8,13E-27	1,45E-54	8,73E-25	9,15E-52
ENSG00000198756	*COLGALT2*	-1,174731	-1,147707	6,49E-11	3,91E-14	1,52E-09	9,23E-13
ENSG00000166165	*CKB*	-1,18113	-2,72807	1,24E-03	1,07E-08	6,42E-03	1,11E-07
ENSG00000122691	*TWIST1*	-1,194682	-1,084083	3,73E-20	3,75E-22	2,28E-18	2,29E-20
ENSG00000163975	*MELTF*	-1,195448	-1,39186	8,65E-19	4,52E-56	4,68E-17	3,05E-53
ENSG00000198440	*ZNF583*	-1,200413	-1,882631	1,08E-04	2,39E-09	7,60E-04	2,75E-08
ENSG00000158859	*ADAMTS4*	-1,218433	-4,189719	1,21E-05	2,05E-87	1,08E-04	3,66E-84
ENSG00000248837	*LOC105374524*	-1,227946	1,723785	9,80E-11	2,71E-09	2,24E-09	3,09E-08
ENSG00000074416	*MGLL*	-1,233486	-2,239167	2,31E-08	1,04E-60	3,53E-07	9,23E-58
ENSG00000154188	*ANGPT1*	-1,233912	-1,373568	1,93E-06	3,07E-07	2,06E-05	2,51E-06
ENSG00000164946	*FREM1*	-1,248028	-1,042277	5,15E-13	9,50E-12	1,58E-11	1,59E-10
ENSG00000197937	*ZNF347*	-1,248827	-1,197475	2,91E-05	1,39E-05	2,38E-04	8,22E-05
ENSG00000260941	*LINC00622*	-1,255234	-1,233008	3,96E-19	3,10E-20	2,22E-17	1,51E-18
ENSG00000170425	*ADORA2B*	-1,292913	-2,111403	7,54E-06	2,66E-13	7,07E-05	5,53E-12
ENSG00000075618	*FSCN1*	-1,303511	-4,058051	1,71E-05	4,84E-73	1,47E-04	5,92E-70
ENSG00000188643	*S100A16*	-1,306377	-1,324938	1,03E-11	1,80E-23	2,74E-10	1,27E-21

ENSG00000166664	*CHRFAM7A*	-1,316353	-1,277891	1,78E-07	5,08E-08	2,34E-06	4,72E-07
ENSG00000175344	*CHRNA7*	-1,323878	-1,204053	6,93E-09	1,91E-08	1,17E-07	1,91E-07
ENSG00000106004	*HOXA5*	-1,329457	-1,158302	1,53E-14	1,35E-12	5,53E-13	2,59E-11
ENSG00000182511	*FES*	-1,392192	-1,272751	1,35E-07	1,98E-07	1,81E-06	1,67E-06
ENSG00000091513	*TF*	-1,404119	-1,419512	1,35E-07	1,16E-07	1,81E-06	1,02E-06
ENSG00000100842	*EFS*	-1,410147	-2,853593	1,59E-03	1,96E-05	7,92E-03	1,12E-04
ENSG00000197106	*SLC6A17*	-1,468864	-1,048252	1,59E-06	3,22E-05	1,72E-05	1,76E-04
ENSG00000205336	*ADGRG1*	-1,492824	-1,221471	3,12E-42	3,18E-25	7,72E-40	2,63E-23
ENSG00000160469	*BRSK1*	-1,50098	-1,737848	9,41E-07	1,58E-10	1,08E-05	2,20E-09
ENSG00000163762	*TM4SF18*	-1,50653	-2,033099	2,02E-08	1,78E-17	3,14E-07	6,21E-16
ENSG00000168389	*MFSD2A*	-1,518262	-1,388231	5,87E-10	2,56E-09	1,21E-08	2,93E-08
ENSG00000118785	*SPP1*	-1,582235	-1,908989	2,05E-06	5,39E-28	2,17E-05	6,11E-26
ENSG00000164949	*GEM*	-1,592914	-1,193433	1,80E-12	5,16E-09	5,22E-11	5,65E-08
ENSG00000136193	*SCRN1*	-1,59382	-2,755628	1,77E-08	1,58E-34	2,77E-07	3,04E-32
ENSG00000126856	*PRDM7*	-1,614506	1,031732	2,26E-37	1,39E-11	4,59E-35	2,29E-10
ENSG00000189144	*ZNF573*	-1,660044	-1,9994	4,73E-08	5,12E-12	6,89E-07	8,98E-11
ENSG00000234444	*ZNF736*	-1,666004	-3,857471	6,02E-08	1,64E-59	8,61E-07	1,34E-56
ENSG00000165209	*STRBP*	-1,683288	-1,107918	2,97E-37	1,06E-18	5,97E-35	4,32E-17
ENSG00000175352	*NRIP3*	-1,696426	1,145184	3,59E-26	2,68E-09	3,63E-24	3,05E-08
ENSG00000167680	*SEMA6B*	-1,701866	-2,246347	6,69E-05	2,82E-07	4,99E-04	2,31E-06
ENSG00000175445	*LPL*	-1,719794	-1,170922	3,48E-39	1,80E-31	7,36E-37	2,80E-29
ENSG00000168079	*SCARA5*	-1,726264	-1,556895	5,65E-06	8,11E-06	5,46E-05	4,97E-05
ENSG00000122547	*EEPD1*	-1,726861	-1,03881	1,72E-11	9,50E-06	4,36E-10	5,76E-05
ENSG00000175356	*SCUBE2*	-1,743945	1,075899	4,54E-12	2,52E-04	1,26E-10	1,13E-03
ENSG00000128564	*VGF*	-1,77774	-1,27088	1,50E-07	5,48E-05	1,99E-06	2,86E-04
ENSG00000005471	*ABCB4*	-1,787413	-1,21881	4,91E-17	5,84E-11	2,27E-15	8,63E-10
ENSG00000197134	*ZNF257*	-1,790952	-4,445527	2,53E-05	1,56E-34	2,10E-04	3,02E-32
ENSG00000234511	*C5orf58*	-1,800509	-1,7154	1,65E-04	5,55E-05	1,10E-03	2,89E-04
ENSG00000010319	*SEMA3G*	-1,839195	-2,180776	1,90E-04	1,00E-05	1,24E-03	6,07E-05
ENSG00000169129	*AFAP1L2*	-1,845812	-1,929762	9,95E-27	4,13E-38	1,05E-24	9,76E-36
ENSG00000157680	*DGKI*	-1,859202	1,122435	9,16E-59	3,29E-13	4,90E-56	6,75E-12
ENSG00000182985	*CADM1*	-1,892726	-1,718074	1,66E-32	1,09E-39	2,50E-30	2,88E-37
ENSG00000171195	*MUC7*	-2,012195	-1,167368	1,53E-36	1,03E-09	2,83E-34	1,26E-08
ENSG00000003249	*DBNDD1*	-2,025566	-2,079571	2,82E-09	1,96E-11	5,07E-08	3,14E-10
ENSG00000153956	*CACNA2D1*	-2,038844	-5,677394	1,59E-04	1,93E-24	1,07E-03	1,49E-22
ENSG00000153208	*MERTK*	-2,155518	-1,346198	7,49E-10	1,74E-05	1,50E-08	1,01E-04
ENSG00000212747	*RTL8B*	-2,15941	-1,039875	8,19E-16	2,28E-05	3,36E-14	1,29E-04
ENSG00000145040	*UCN2*	-2,159859	-1,124571	1,81E-43	7,85E-12	4,84E-41	1,33E-10
ENSG00000116962	*NID1*	-2,190675	-3,168104	4,95E-63	2,44E-197	2,87E-60	4,79E-193
ENSG00000106537	*TSPAN13*	-2,260637	-1,070944	1,05E-57	3,26E-12	5,31E-55	5,86E-11
ENSG00000162409	*PRKAA2*	-2,305861	-1,106255	2,47E-27	1,75E-07	2,73E-25	1,49E-06
ENSG00000162817	*C1orf115*	-2,370661	-1,057151	3,99E-83	9,21E-12	3,86E-80	1,55E-10
ENSG00000204147	*ASAH2B*	-2,395787	-1,48149	1,18E-32	6,23E-14	1,82E-30	1,42E-12
ENSG00000188611	*ASAH2*	-2,46093	-1,450237	1,21E-30	5,71E-12	1,60E-28	9,95E-11
ENSG00000163273	*NPPC*	-2,520817	-1,288799	9,72E-09	7,93E-05	1,60E-07	3,98E-04
ENSG00000078098	*FAP*	-2,574475	-2,186742	1,89E-28	3,29E-21	2,16E-26	1,79E-19
ENSG00000131849	*ZNF132*	-2,579265	-1,368464	1,20E-04	6,14E-04	8,31E-04	2,50E-03
ENSG00000170891	*CYTL1*	-2,610459	-4,399968	7,25E-05	1,19E-10	5,35E-04	1,68E-09
ENSG00000183098	*GPC6*	-2,612445	-4,381227	1,79E-09	8,52E-40	3,34E-08	2,29E-37
ENSG00000152217	*SETBP1*	-2,654771	-3,411923	2,54E-04	1,11E-05	1,61E-03	6,63E-05
ENSG00000186472	*PCLO*	-2,712865	-1,191557	1,59E-06	8,30E-04	1,73E-05	3,26E-03
ENSG00000243137	*PSG4*	-2,743556	-1,574162	3,08E-06	1,73E-04	3,16E-05	8,06E-04
ENSG00000101115	*SALL4*	-2,84213	-1,907139	1,33E-07	1,64E-05	1,79E-06	9,55E-05
ENSG00000205611	*LOC107985433*	-2,854752	-1,072017	1,91E-06	1,58E-03	2,05E-05	5,80E-03
ENSG00000140522	*RLBP1*	-2,872886	1,018803	3,37E-59	5,20E-07	1,85E-56	4,06E-06
ENSG00000146674	*IGFBP3*	-2,885776	-1,865919	9,80E-91	1,35E-27	1,42E-87	1,45E-25
ENSG00000077522	*ACTN2*	-2,955289	-1,411778	2,26E-17	1,75E-05	1,08E-15	1,02E-04
ENSG00000159164	*SV2A*	-3,008189	-2,9302	8,91E-80	3,38E-67	7,86E-77	3,68E-64
ENSG00000121966	*CXCR4*	-3,0577	-2,190217	4,69E-10	2,51E-07	9,77E-09	2,08E-06
ENSG00000182247	*UBE2E2*	-3,061495	-2,182342	1,75E-19	4,96E-11	1,01E-17	7,44E-10
ENSG00000152582	*SPEF2*	-3,143853	-1,889504	1,08E-16	2,98E-07	4,82E-15	2,44E-06
ENSG00000169297	*NR0B1*	-3,148579	-1,246468	3,37E-32	3,94E-06	4,95E-30	2,56E-05
ENSG00000135424	*ITGA7*	-3,193158	-1,560461	3,93E-109	1,88E-19	1,14E-105	8,26E-18

ENSG00000163568	*AIM2*	-3,247561	-1,453561	3,76E-12	1,56E-04	1,05E-10	7,35E-04
ENSG00000227051	*C14orf132*	-3,266287	-5,363855	1,12E-04	1,43E-07	7,82E-04	1,23E-06
ENSG00000065923	*SLC9A7*	-3,305648	-3,277593	6,51E-52	4,33E-45	2,64E-49	1,49E-42
ENSG00000141934	*PLPP2*	-3,342734	-1,281141	1,72E-20	7,26E-05	1,08E-18	3,68E-04
ENSG00000138772	*ANXA3*	-3,392527	-1,284772	5,94E-77	6,36E-13	4,83E-74	1,26E-11
ENSG00000162723	*SLAMF9*	-3,455149	-2,908324	1,12E-25	4,65E-19	1,10E-23	1,96E-17
ENSG00000096696	*DSP*	-3,457885	-2,479969	2,66E-48	6,57E-22	9,48E-46	3,91E-20
ENSG00000102452	*NALCN*	-3,571898	-1,352393	5,70E-15	2,49E-04	2,13E-13	1,12E-03
ENSG00000157404	*KIT*	-3,588105	-1,816634	2,43E-11	3,66E-05	6,06E-10	1,98E-04
ENSG00000036565	*SLC18A1*	-3,743016	-1,878356	1,61E-35	2,26E-08	2,77E-33	2,23E-07
ENSG00000137642	*SORL1*	-3,759267	-1,357559	1,22E-159	1,09E-10	8,24E-156	1,54E-09
ENSG00000117122	*MFAP2*	-3,877072	-2,702465	1,09E-13	1,19E-07	3,59E-12	1,05E-06
ENSG00000105825	*TFPI2*	-3,909761	-2,947398	1,39E-08	1,55E-06	2,23E-07	1,09E-05
ENSG00000198223	*CSF2RA*	-3,947053	-5,93487	1,22E-46	1,76E-149	3,75E-44	1,73E-145
ENSG00000198682	*PAPSS2*	-3,971837	-1,416942	9,54E-76	1,59E-08	7,17E-73	1,60E-07
ENSG00000131089	*ARHGEF9*	-4,000428	1,200237	1,29E-46	1,03E-03	3,92E-44	3,94E-03
ENSG00000143340	*FAM163A*	-4,050096	-3,556989	3,56E-116	1,51E-90	1,45E-112	4,24E-87
ENSG00000162458	*FBLIM1*	-4,167646	-4,840634	9,96E-17	1,53E-25	4,48E-15	1,32E-23
ENSG00000091490	*SEL1L3*	-4,266895	-2,628372	3,88E-84	2,76E-28	4,04E-81	3,24E-26
ENSG00000067177	*PHKA1*	-4,36531	-2,565095	4,37E-108	5,43E-27	1,11E-104	5,45E-25
ENSG00000109956	*B3GAT1*	-4,447017	-5,767819	1,83E-07	9,53E-13	2,39E-06	1,86E-11
ENSG00000116132	*PRRX1*	-4,704198	-1,111526	4,95E-40	5,65E-04	1,09E-37	2,32E-03
ENSG00000165929	*TC2N*	-5,354045	-1,063314	2,69E-51	8,49E-04	1,05E-48	3,32E-03
ENSG00000152128	*TMEM163*	-5,532916	-3,238019	8,05E-29	2,16E-09	9,44E-27	2,51E-08
ENSG00000169862	*CTNND2*	-5,8665	-1,259626	4,71E-12	8,18E-04	1,30E-10	3,22E-03
ENSG00000100077	*GRK3*	-5,883616	-2,520245	3,93E-49	3,87E-08	1,42E-46	3,66E-07
ENSG00000198795	*ZNF521*	-6,286324	-1,796798	1,56E-20	1,50E-04	9,84E-19	7,09E-04
ENSG00000166250	*CLMP*	-6,426356	-4,75846	1,88E-08	7,54E-07	2,94E-07	5,69E-06
ENSG00000046604	*DSG2*	-8,419309	-9,36443	3,11E-14	1,18E-18	1,08E-12	4,80E-17
