# Supplementary material for: MX2 mediates establishment of interferon response profile, regulates XAF1, and can sensitize melanoma cells to targeted therapy
Source: Cancer Med. 2021 Mar 18;10(8):2840–54. doi: 10.1002/cam4.3846 (PMC8026919; doi:10.1002/cam4.3846)
Supplement: Supplementary file 14 [file CAM4-10-2840-s006.docx]

**Supporting Information**

**MX2 mediates establishment of interferon response profile, regulates XAF1 and can sensitize melanoma cells to targeted therapy**

Marina Juraleviciute, Jérémie Nsengimana, Julia Newton-Bishop, Gert Jan Hendriks, Ana Slipicevic

**Suppporting Figure legends**

**Suppporting Tables**

Supporting Table 1. Commercial melanoma cell lines used in a study.

Supporting Table 2. Differentially expressed genes between WM983b MX2-overexpressing (MX2) and both GFP-overexpressing (GFP) and untransduced control (CTR).

**Supporting Figure legends**

**Supporting Figure 1. MX2 overexpression downregulates DSG2.** qRT-PCR analysis of *DSG2* expression in WM983b and MM5 (untransduced (CTR) and transduced to overexpress GFP and MX2). Each circle on histogram depicts mean value of an independent experiment and each bar represents the mean +SD (n=3); n.d. – not detected. *DSG2* mRNA expression is normalized to untransduced control of corresponding cell line. Statistical significance was assessed by one-way ANOVA with Tukey’s multiple comparison test after log transformation of the data.

**Supporting Figure 2. Correlation of MX2 and XAF1 expression. a)** *MX*2 and *XAF1* RNA expression correlation in our panel of 45 patient derived lymph node metastatic melanoma samples. Association tested with Pearson’s correlation. **b)** *MX2* and *XAF1* RNA expression correlation in 55 melanoma cell lines from Cancer Cell Line Encyclopedia. Association tested with Pearson’s correlation. **c)** Immunoblot analysis of phosphorylated STAT1 at Y701 and total STAT1 expression in a panel of cell lines from Figure 3b. *Immunoblot of XAF1 protein levels is from Figure 3b provided for comparison. β-actin used for loading control of STAT1 proteins.

**Supporting Figure 3. XAF1 is a downstream target of MX2. a)** Before-after graph depicts changes in growth rates of WM1366 cells after *XAF1* downregulation with siRNA for 72 hrs (n=3). **b)** Proliferation assessment by Incucyte in MM5, WM35, MM388 and WM852 melanoma cells treated with scrambled RNA (siCTR) and *XAF1* specific siRNA for 48 hours. Growth rates were calculated by normalizing cell occupied surface area at a given time point to initial. Each point of growth curves represents mean value ±SD (n=3). Comparison of siCTR and siXAF1 growth rates at 48 hrs time point was performed by Welch’s *t*-test after log transformation of the data. **c)** MM382 and WM1366 melanoma cells were treated with scrambled RNA (siCTR) and *XAF1* specific siRNA for 72 hours and immunoblot analysis was performed to assess XAF1 downregulation effect on MAPK signaling pathway. β-tubulin was used for loading control. **d)** WM983b and WM1366 cells were transfected with control siRNA (siCTR) or *MX2* specific siRNAs (siMX2_1 and siMX2_2) or **e)** *XAF1* specific siRNA (siXAF1) for 24 hours before addition of 25000 IU/mL IFNα for 48 hours and then assessed for MX2 and XAF1 expression by immunoblotting. β-tubulin used for loading control.

**Supporting Figure 4. Association of *XAF1* mRNA expression with Breslow thickness.** *XAF1* mRNA expression correlation with Breslow thickness of primary melanomas in the LMC. Association tested with Pearson’s correlation.

**Supporting Figure 5.** Original immunoblots of MX2, XAF1, IRF1 and H3 used in the **Figure 1d**.

**Supporting Figure 6. a)** Original immunoblots of MX2, pSTAT1 Y701, STAT1 and β-actin used in the **Figure 2a**. **b)** Original immunoblots of MX2, pSTAT1 Y701, STAT1, IRF1, XAF1 and β-actin used in the **Figure 2b**. c) Original immunoblots of MX2, pSTAT1 Y701, STAT1, c-MYCand β-tubulin used in the **Figure 2c**.

**Supporting Figure 7. a)** Original immunoblots of MX2, XAF1 and β-tubulin used in the **Figure 3a**. **b)** Original immunoblots of MX2, XAF1 and β-tubulin used in the **Figure 3b. c)** Original immunoblots of MX2, XAF1, pSTAT1 Y701, STAT1 and β-actin used in the **Figure 3e.**

**Supporting Figure 8. a)** Original immunoblots of MX2, XAF1, pAKT s473, AKT and β-tubulin used in the **Figure 4a**. **b)** Original immunoblots of MX2, XAF1, pAKT s473, AKT and β-tubulin used in the **Figure 4b**.

**Supporting Figure 9. a)** Original immunoblots of MX2, XAF1, pERK ½, ERK, cleaved caspase 3 and β-tubulin used in the **Figure 6b**. **b)** Original immunoblots of MX2, XAF1, pERK ½, ERK, cleaved caspase 3 and β-tubulin used in the **Figure 6d.**

**Supporting Figure 10.** Original immunoblots of pSTAT1 Y701, STAT1 and β-actin used in the **Supporting Figure 2c.**

**Supporting Figure 11. a)** Original immunoblots of MX2, pERK1/2, ERK, XAF1 and β-tubulin used in the **Supporting Figure 3c. b)** Original immunoblots of MX2, XAF1 and β-tubulin used in the **Supporting Figure 3d. c)** Original immunoblots of MX2, XAF1 and β-tubulin used in the **Supporting Figure 3e.**
